# Causal Relationship Between Sjögren’s Syndrome and Gut Microbiota: A Two-Sample Mendelian Randomization Study

**DOI:** 10.3390/biomedicines12102378

**Published:** 2024-10-18

**Authors:** Xinrun Wang, Minghui Liu, Weiping Xia

**Affiliations:** 1 Department of Critical Care Medicine, Xiangya Hospital, Central South University, Changsha 410008, China; wangxr1893@csu.edu.cn; 2 National Clinical Research Center for Geriatric Disorders, Xiangya Hospital, Central South University, Changsha 410008, China; liuminghui199710@163.com; 3 Department of Urology, Xiangya Hospital, Central South University, Changsha 410008, China

**Keywords:** gut microbiota, Sjögren’s syndrome, immune related diseases, Mendelian randomization

## Abstract

**Background:** Gut microbiota have been previously reported to be related to a variety of immune diseases. However, the causal connection between Sjögren’s syndrome (SS) and gut microbiota has yet to be clarified. **Methods:** We employed a two-sample Mendelian randomization (MR) analysis to evaluate the causal connection between gut microbiota and SS, utilizing summary statistics from genome-wide association studies (GWASs) obtained from the MiBioGen and FinnGen consortia. The inverse variance weighted (IVW) approach represents the primary method of Mendelian randomization (MR) analysis. Sensitivity analysis was used to eliminate instrumental variables heterogeneity and horizontal pleiotropy. In addition, we performed an analysis using independent GWAS summary statistics for SS from the European Bioinformatics Institute (EBI) dataset for further verify our results. **Results:** IVW results demonstrated that the phylum *Lentisphaerae* (OR = 0.79, 95% CI: 0.63–0.99, *p* = 0.037), class *Deltaproteobacteria* (OR = 0.67, 95% CI: 0.47–0.96, *p* = 0.030), family *Porphyromonadaceae* (OR = 0.60, 95% CI: 0.38–0.94, *p* = 0.026), genus *Eubacterium coprostanoligenes* group (OR = 0.61, 95% CI: 0.4–0.93, *p* = 0.021), genus *Blautia* (OR = 0.62, 95% CI: 0.43–0.90, *p* = 0.012), genus *Butyricicoccus* (OR = 0.61, 95% CI: 0.42–0.90, *p* = 0.012), genus *Escherichia.Shigella* (OR = 0.7, 95% CI: 0.49–0.99, *p* = 0.045) and genus *Subdoligranulum* (OR = 0.61, 95% CI: 0.44–0.86, *p* = 0.005) exhibited protective effects on SS. Relevant heterogeneity of horizontal pleiotropy or instrumental variables was not detected. Furthermore, repeating our results with an independent cohort provided by the EBI dataset, only the genus *Eubacterium coprostanoligenes* group remained significantly associated with the protective effect on SS (OR = 0.41, 95% CI: 0.18–0.91, *p* = 0.029). Two-step MR analysis further revealed that genus *Eubacterium coprostanoligenes* group exerts its protective effect by reducing CXCL6 levels in SS (OR, 0.87; 95% CI = 0.76–0.99, *p* = 0.033). **Conclusions:** Our study using two-sample MR analysis identified a causal association between multiple genera and SS. A two-step MR result calculated that genus *Eubacterium coprostanoligenes* group mediated its protective effect by reducing CXCL6 levels in SS. However, the datasets available from the MiBioGen and FinnGen consortia do not provide sufficient information or comprehensive demographic data for subgroup analyses. Additional validation using various omics technologies is necessary to comprehend the development of SS in the intricate interplay between genes and the environment over a period of time.

## 1. Introduction

Sjögren’s syndrome (SS) is a chronic inflammatory autoimmune disease primarily affecting the exocrine glands such as the lacrimal and salivary glands. It is characterized by lymphocytic infiltrates in the affected glands, also referred to as autoimmune exocrine gland epithelitis or autoimmune exocrine disease [1,2]. SS is one of the most prevalent autoimmune disorders [3]. The first case of SS is generally believed to have been described by Mikulicz, J.H. in 1892 [4]. SS is always classified into primary SS and secondary SS. According to the results of worldwide epidemiological studies, the incidence of SS is 6.92 cases per 100,000 person-years, and the population prevalence is approximately 60.82 cases per 100,000 inhabitants [5]. The disease predominantly affects females (with a female to male ratio of nine to one) and its impact is often more severe in women [6]. Patients with SS typically endure persistent and severe pain, alongside various physical symptoms including decayed teeth, keratoconjunctivitis sicca, xerostomia, vaginal dryness, and musculoskeletal pain [7,8,9]. These symptoms contribute to a significant social and medical burden and adversely affect the patients’ quality of life. While researchers speculate that SS may arise from the interaction of genetic, hormonal, and environmental factors, leading to the activation of epithelial cells, the precise etiopathogenesis of SS remains largely unclear [8,10]. Hence, further exploration of SS’s underlying mechanisms and the development of novel therapeutic targets are essential to enhance the effectiveness of treatment strategies.

Gut microbiota, the most complex and largest community of microorganisms residing within the human body, play an important role in shaping the development and maintenance of the host’s health through various interactions with the host such as co-metabolism of substrates, metabolites derived from the gut microbiota and immune regulation [11,12,13]. Recent research has widely reported the crosstalk between the gut microbiome and immune diseases [12,14,15]. For instance, the maintenance of immune balance and human health largely depends on the stability of the gut microbiota. Numerous immune-related inflammatory disorders, such as inflammatory bowel diseases, diabetes mellitus, and systemic lupus erythematosus (SLE), are intricately linked to alterations in the gut microbiota and its metabolites [14,16]. Some studies have shown that the abundance of *Collinsella* and *Akkermansia* are increased in some very active rheumatoid arthritis (RA) patients [17], while in an early RA patient, the abundance of *Bacteroidetes* are decreased [18]. Furthermore, as the incidence of autoimmune diseases rises, the gut microbiome composition has been associated with the pathogenesis of SLE. Tomofuji Y et al. found that *Streptococcus anginosus* and *Streptococcus intermedius* were enriched in Japanese patients with SLE [19]. Additionally, *Clostridium species ATCC BAA-442*, *Clostridium leptum*, *Shuttleworthia satelles*, *Bacteroides fragilis*, *Actinomyces massiliensis*, and *Atopobium rimae* were found to be increased in the gut microbiota of SLE patients [20]. Moreover, accumulating evidence strongly supports that the effect of the gut microbiota on the immune system plays a crucial role in the development and course of multiple sclerosis (MS) [15,21,22]. Compared with healthy controls, *Akkermansia muciniphila*, *Ruthenibacterium lactatiformans*, *Hungatella hathewayi*, and *Eisenbergiella tayi* were upregulated, while *Faecalibacterium prausnitzii* and *Blautia species* were downregulated in MS patients [23]. Accordingly, targeted antibiotics treatment, fecal microbiota transplantation, and individual diet-based therapies have shown encouraging outcomes in preventing autoimmune disorders [24,25]. However, researchers have a limited understanding of the microbiota in individuals with SS, and there are few instances of metagenomic studies providing a detailed analysis of the gut microbiota in SS. Research findings observed that primary SS patients have been found to have a lower abundance and evenness in their gut microbiota, as well as a significantly different community distribution, compared to healthy controls [26]. Shotgun metagenomic sequencing results showed that the fecal samples from patients with primary SS carried more virulence genes [26]. Through 16S ribosomal RNA gene sequencing of the fecal samples from primary SS patients, the results showed that gut microbiota presented a higher abundance of *Bacteroidota* and a lower *Firmicutes*/*Bacteroidota* ratio in the primary SS patients [27]. The literature about the biological mechanisms and gut microbiota in SS are also very rare. A study by Woo, J.S. et al. found that *Lactobacillus acidophilus* and propionate may improve the occurrence and progression of SS by inhibiting the STIM 1-STING signaling pathway [28]. The causal connection between the gut microbiome and SS remains uncertain; therefore, investigating the potential causal relationship between the gut microbiota and SS could offer new avenues for preventing and treating SS.

Mendelian randomization (MR) studies, similar to randomized controlled trials, are a novel method for investigating the causal relationships between exposures and disease outcome [29]. A two-sample MR analysis leverages the data on single-nucleotide polymorphisms (SNPs) associated with the exposure and disease outcomes from separate genome-wide association studies (GWASs) to produce a combined causal estimation [30]. SNPs function based on the basic principle of randomly distributing genetic variations during meiosis, which help to minimize the influence of potential confounding factors and lower the likelihood of reverse causation, as the genetic variations occur before the onset of disease [31]. MR has been extensively utilized to investigate the possible causal relationships between the gut microbiota and autoimmune diseases [32,33,34]. MiBioGen provides comprehensive gut microbiota datasets from multiple populations, with microbiota analysis conducted using standardized methods. FinnGen offers extensive health data, including GWAS summary statistics rigorously adjusted for potential confounding factors such as age, sex, and genetic typing batches. To the best of our knowledge, there are no available GWASs specifically for well-classified primary or secondary SS patients. We recognize that this may be a potential source of heterogeneity in real-world clinical populations. Hence, we used a two-sample MR analysis to explore the potential causal impact of the gut microbiota on SS by utilizing the largest summary statistics from GWASs carried out by the MiBioGen and FinnGen consortia.

## 2. Methods

### 2.1. Exposure Data

In our research, we chose SNPs that are highly correlated with the makeup of the human gut microbiome to serve as instrumental variables. These SNPs were derived from a large scale GWAS conducted by the international consortium MiBioGen, which analyzed 16S rRNA gene sequencing data from 18,340 samples across 24 cohorts representing diverse countries. In total, 211 gut microbiome taxa, comprising 131 genera, 35 families, 20 orders, 16 classes, and 9 phyla, were included in the analysis. Furthermore, a comprehensive proteomic study was conducted to derive summary statistics of genetic associations with 91 inflammatory factors [35].

### 2.2. Results Information

Briefly, GWAS summary statistics for SS were obtained from the 7th release of the FinnGen consortium, including 1981 cases and 300,162 controls, with adjustments made for age, sex, 10 principal components, and genotyping batches. The diagnosis criteria of SS were based on the M35.0 codes in the *International Classification of Diseases, 10th Revision* (ICD-10). ICD-10 does not differentiate between primary and secondary SS (i.e., associated with other autoimmune diseases such as RA or SLE) under M35.0 [36,37]. In addition, van der Meulen, T.A. et al. found that the gut microbiome of primary SS and SLE patients is similar, but there is a difference in the composition of the oral microbiome [38]. Unfortunately, our current dataset does not include a specific disease control group (e.g., RA or SLE patients). Therefore, to enhance the credibility of our findings, we conducted an extra analysis using independent GWAS summary data for SS from the European Bioinformatics Institute (EBI) dataset (ebi-a-GCST90013879), which comprised 407,746 individuals and was used to replicate our results.

### 2.3. Instrumental Variable Selection

The accuracy and credibility of causal inferences heavily rely on the careful selection of instrumental variables. In order to achieve a more thorough outcome, SNPs linked to the gut microbiome and inflammatory factors were examined as potential instrumental variables at the genome-wide significance threshold (*p* < 1 × 10^−5^). To maintain statistical independence between SNPs, we performed a linkage disequilibrium analysis (R2 < 0.001) using a clumping window size of 10,000 kb, utilizing data from the European-based 1000 Genome Projects [39]. In addition, the F-statistic was utilized to assess the robustness of the chosen SNPs and to identify any potential instrumental bias (F = beta^2^/se^2^). An F-statistic greater than 10 was considered to suggest that weak instrumental variables did not significantly contribute to bias [32]. Finally, in order to minimize the association between SNPs and potential confounding variables, we applied PhenoScanner (https://ldlink.nih.gov/?tab=ldtrait, accessed on 1 October 2022) to examine all included instrumental variables, and then we excluded those linked to potential confounding factors.

### 2.4. Statistical Analysis

We employed five highly efficient and widely used MR techniques to examine the potential causal link between gut microbiota and SS, including the inverse-variance weighted (IVW) method, MR-Egger regression, weighted mode, weighted median, and simple mode [40]. IVW, the primary method for MR analysis, was employed to infer causality. IVW operates similarly to a meta-analysis framework, translating the causal influence of instrumental variables on exposure into a weighted regression in order to acquire an overall estimate of the impact of gut microbiome on SS risk. MR-Egger regression assesses whether instrumental variables have horizontal pleiotropy on results and calculates causal inferences, which always arise when the instrumental variables impact the outcomes through pathways that are not influenced by the exposure. Additionally, MR-PRESSO analysis identifies and addresses horizontal pleiotropy by excluding data points that may be influencing the results. After that, the MR-PRESSO method uses the remaining instrumental variables to re-estimate the causal effect. Compared with the MR-Egger method, the MR-PRESSO analysis has more precision and statistical power in MR studies. The Cochran’s Q test was adopted to assess the degree of heterogeneity among SNPs included in each microbial taxon. The scatter plots and funnel plots were generated to visualize MR analyses outcomes and screen out potential outliers. Leave-one-out analysis was performed to detect the potential heterogeneity of SNPs that could impact the outcomes through unknown pathways [41]. This approach was used to estimate the impact of a single SNP from the overall set of instrumental variables. In this research, the statistical analyses for all MRs were performed using R software, version 4.1.3, along with the “Two Sample MR” R package and the “MRPRESSO” R package.

We utilized a two-step MR method to investigate the potential mediating role of inflammatory factors on the association between gut microbiota and SS. First, we estimated the direct impact of genetically predicted gut microbiota taxa on SS (denoted as α) using Univariable Mendelian Randomization (UVMR), determining the overall association between gut microbiota and SS. Next, UVMR was used to identify inflammatory factors significantly associated with SS. We selected these factors as potential mediators in the pathway between gut microbiota and SS. We then applied Multivariable Mendelian Randomization (MVMR) to account for the genetic impacts of gut microbiota and evaluate the independent influence of the identified inflammatory factors on SS (denoted as β2). To assess the potential impact of the gut microbiota taxa on the identified inflammatory factors, UVMR was performed to assess the association between gut microbiota and these inflammatory mediators (denoted as β1). Finally, we calculated the mediation effect ratio as β1 * β2/α. This ratio quantified the extent to which the protective effect of GM on SS could be attributed to the modulation of inflammatory factors.

## 3. Results

### 3.1. SNPs Selection

After several quality control steps, we identified 102, 178, 215, 375, and 1381 SNPs (*p* < 1 × 10^−5^) at the phylum, class, order, family, and genus levels. In total, we selected 2251 SNPs as the instrumental variables. The F-statistics of all the instrumental variables exceeded 10, suggesting no indication of weak instrument bias.

### 3.2. MR Analyses

**Figure 1:**
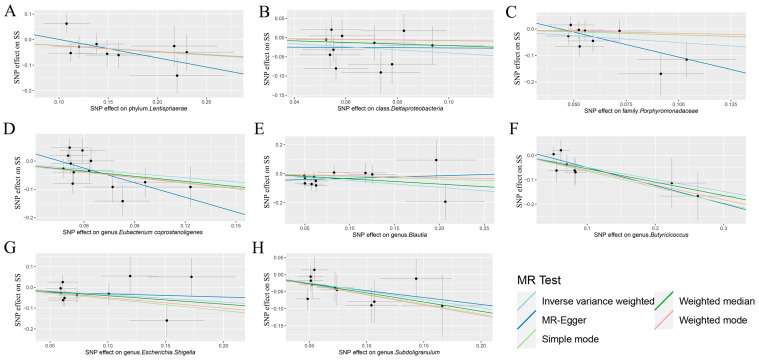
Scatter plots comparing the genetic correlations between gut microbiota and SS using various MR techniques. (**A**) The impact of the phylum *Lentisphaerae* on SS. (**B**) The impact of the class *Deltaproteobacteria* on SS. (**C**) The impact of the family *Porphyromonadaceae* on SS. (**D**) The impact of the genus *Eubacterium coprostanoligenes* on SS. (**E**) The impact of the genus *Blautia* on SS. (**F**) The impact of the genus *Butyricicoccus* on SS. (**G**) The impact of the genus *Escherichia.Shigella* on SS. (**H**) The impact of the genus *Subdoligranulum* on SS. The inclines of the line indicate the individual causal impact of each method. MR: Mendelian randomization; SS: Sjögren’s syndrome.

The IVW analysis showed that the phylum *Lentisphaerae*, class *Deltaproteobacteria*, family *Porphyromonadaceae*, genus *Eubacterium coprostanoligenes* group, genus *Blautia*, genus *Butyricicoccus*, genus *Escherichia.Shigella*, and genus *Subdoligranulum* were associated with SS. The phylum *Lentisphaerae* (OR = 0.79, 95% CI: 0.63–0.99, *p* = 0.037), class *Deltaproteobacteria* (OR = 0.67, 95% CI: 0.47–0.96, *p* = 0.030), family *Porphyromonadaceae* (OR = 0.60, 95% CI: 0.38–0.94, *p* = 0.026), genus *Eubacterium coprostanoligenes* group (OR = 0.61, 95% CI: 0.4–0.93, *p* = 0.021), genus *Blautia* (OR = 0.62, 95% CI: 0.43–0.90, *p* = 0.012), genus *Butyricicoccus* (OR = 0.61, 95% CI: 0.42–0.90, *p* = 0.012), genus *Escherichia.Shigella* (OR = 0.7, 95% CI: 0.49–0.99, *p* = 0.045), and genus *Subdoligranulum* (OR = 0.61, 95% CI: 0.44–0.86, *p* = 0.005) had protective effects on SS (Table 1). The causal relationship between the gut microbiota and SS is depicted in Figure 1 using scatter plots. The instrumental variables specifically utilized in the MR analysis are detailed in Table S1. Table S2 presents the complete findings from all the MR analyses that were performed. The EBI dataset provided an independent cohort to replicate our results. In this replication analysis, only the genus *Eubacterium coprostanoligenes* group remained significantly associated with a protective effect on SS (OR = 0.41, 95% CI: 0.18–0.91, *p* = 0.029) (Table S6 and Supplementary Figure S1). This validation strengthens the robustness of our findings and enhances the external validity of our conclusions, particularly for the *Eubacterium coprostanoligenes* group, which consistently demonstrated a defensive role in both the FinnGen and EBI datasets.

### 3.3. Sensitivity Analyses

**Figure 2:**
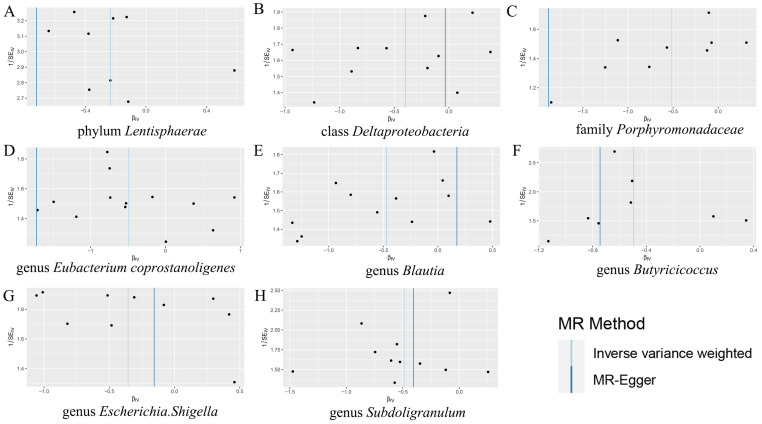
Funnel plots used to evaluate the pleiotropy of observed causal connections between gut microbiota and SS for (**A**) phylum *Lentisphaerae*; (**B**) class *Deltaproteobacteria*; (**C**) family *Porphyromonadaceae*; (**D**) genus *Eubacterium coprostanoligenes*; (**E**) genus *Blautia*; (**F**) genus *Butyricicoccus*; (**G**) genus *Escherichia.Shigella*; and (**H**) genus *Subdoligranulum*.

**Figure 3:**
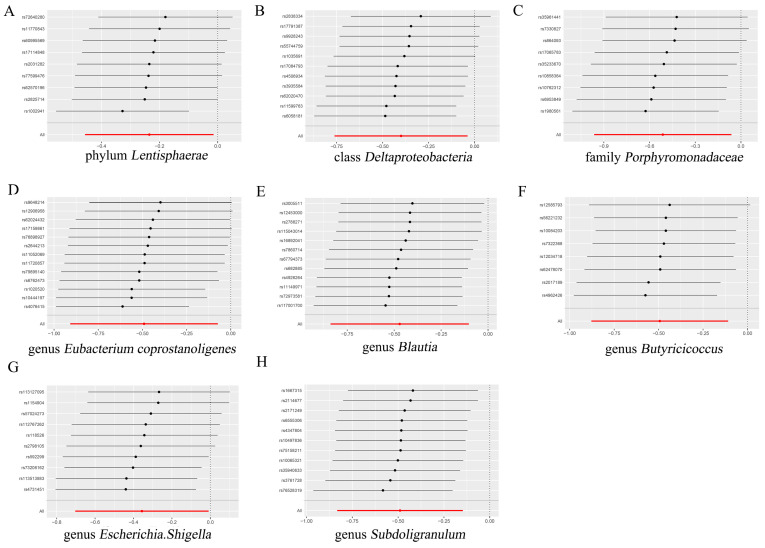
Leave-one-out analysis allowed us to assess the impact of individual SNPs on the relationship between gut microbiota and SS risk. (**A**) phylum *Lentisphaerae*; (**B**) class *Deltaproteobacteria*; (**C**) family *Porphyromonadaceae*; (**D**) genus *Eubacterium coprostanoligenes*; (**E**) genus *Blautia*; (**F**) genus *Butyricicoccus*; (**G**) genus *Escherichia.Shigella*; and (**H**) genus *Subdoligranulum*. This approach demonstrates how each specific SNP affects the overall result. SNPs, Single nucleotide polymorphism; SS: Sjögren’s syndrome.

Based on the findings from Cochran’s Q test, no statistically significant diversity was detected among these instrumental variables (Table S3). An analysis of the MR-Egger regression intercept suggested that there is no indication of directional horizontal pleiotropy in the association between gut microbiota and SS (Table S4). A visual examination of the scatter plots revealed a better sensitivity between the instrumental variables and more stable outcomes (Figure 2). The leave-one-out plots further supported the robustness of the outcomes in this MR analysis, as omitting any single SNP did not fundamentally alter the outcomes (Figure 3). Furthermore, the MR-PRESSO analysis did not identify any anomalies in the findings (Table S5). Consequently, the existing evidence did not provide backing for the existence of horizontal pleiotropy in the association between these bacteria and SS.

### 3.4. Mediation Analysis Results

The initial analysis from the FinnGen GWAS, along with the replication results from the EBI dataset, consistently demonstrated that the genus *Eubacterium coprostanoligenes* group has a protective effect on SS. Specifically, higher levels of C-X-C motif chemokine 11 (OR = 1.24, 95% CI = 1.05–1.48, *p* = 0.013), C-X-C motif chemokine 6 (CXCL6, OR = 1.51; 95% CI = 1.04–2.18, *p* = 0.029), and Interleukin-13 (OR = 1.22, 95% CI = 1.02–1.47, *p* = 0.033) were linked to an increased risk of SS. In contrast, elevated levels of CD40L receptor (OR = 0.85, 95% CI = 0.74–0.97, *p* = 0.019), Cystatin D (OR = 0.75, 95% CI = 0.59–0.96, *p* = 0.022), Monocyte chemoattractant protein-3 (OR = 0.79, 95% CI = 0.66–0.95, *p* = 0.011), Tumor necrosis factor (OR = 0.76, 95% CI = 0.61–0.95, *p* = 0.017), Tumor necrosis factor ligand superfamily member 14 (OR = 0.86, 95% CI = 0.74–0.99, *p* = 0.043), and Interleukin-2 (OR = 0.71, 95% CI = 0.52–0.98, *p* = 0.039) were associated with a reduced risk of SS, as demonstrated by the IVW method (Table S7). To investigate how inflammatory factors mediate the connection between the gut microbiota and SS, we first assessed whether the genus *Eubacterium coprostanoligenes* group was linked to the nine identified inflammatory factors (Table S8). Our analysis showed a significant association between the genus *Eubacterium coprostanoligenes* group and a reduced likelihood of elevated CXCL6 levels (OR = 0.87, 95% CI = 0.76–0.99, *p* = 0.033). A mediation analysis demonstrated that the genus *Eubacterium coprostanoligenes* group mediated approximately 7.94% of its protective effect on SS by reducing CXCL6 levels (Supplementary Figure S1).

## 4. Discussion

Autoimmune diseases, including SLE, SS, RA, MS, and others, are a category of illnesses that arise when the body’s immune system erroneously targets its own tissues. Nowadays, although genetic components, environmental influences, and their interplay are acknowledged as major contributors to the development of autoimmune diseases, there is growing evidence indicating that changes in the gut microbiota composition are closely associated with these conditions [32,42]. To our understanding, our research is the first MR analysis to explore the potential causal link between the gut microbiota and SS. Our sensitivity analyses showed that the IVW method identified one phylum, one class, one family, and five genuses with significant results. We also conducted sensitivity analyses using different approaches, such as the weighted median, simple mode, and MR-Egger regression, to account for potential pleiotropy and evaluate the reliability of our results. The weighted median method identified only three genera (genus *Eubacterium coprostanoligenes* group, genus *Butyricicoccus*, and genus *Subdoligranulum*) that are significantly involved in the pathogenesis of SS. Therefore, we have chosen the IVW method as our main analysis due to its high statistical power and efficiency. Our results showed that variations in the composition of the gut microbiota from the phylum to the genus level between the healthy controls and SS patients. These differences in microbial diversity and categorization indicate a potential link between the gut microbiota and the development of SS.

The relationship between these differentially expressed flora identified in our results and SS has been scarcely documented. However, the risk-associated microbiota have frequently been connected with various autoimmune diseases or inflammatory-related diseases. For example, the phylum *Lentisphaerae* was elevated in the fecal samples from systemic sclerosis patients and early-stage silicosis patients [43,44], while *Lentisphaerae* levels were notably decreased in autoimmune hepatitis patients and weaned piglets [45,46]. Researchers also suggested that the decrease in *Lentisphaerae* in the fecal samples may be related to weight loss in the weaned piglets [46]. The presence of the class *Deltaproteobacteria* in the gut microbiome has been identified as a significant risk factor, with its higher abundance playing a key role in the development of Graves’ disease and chronic kidney diseases [39,47]. Conversely, the abundance of the class *Deltaproteobacteria* and its descendants were significantly lower in T1DM-onset pediatric patients, suggesting a link to iron or carbon metabolism [48,49]. Similarly, the dysregulation of the *Porphyromonadaceae* family expression is linked to autoimmune diseases, including down-regulation in type 2 diabetes and up-regulation in patients with atopic dermatitis or ankylosing spondylitis [50,51,52]. Moreover, the gut microbiota showed variations in the expression of *Porphyromonadaceae* between genders, potentially influencing the differences in immune system responses [53,54]. The *Eubacterium coprostanoligenes* group, a type of gram-negative bacteria, are known to regulate immune responses through the production of butyrate [55]. Animal experiments by Ying He have reported that Danggui Sini decoction may contribute to the relief of rheumatoid arthritis by affecting three key gut microbiomes (including the *Eubacterium coprostanoligenes* group) and their metabolites [56]. Another study demonstrated that the *Eubacterium coprostanoligenes* group was abundant in IgA nephropathy, and it was positively correlated with L-tryptophan levels and BUN levels [57]. *Blautia*, a type of anaerobic bacteria known for its probiotic properties, is commonly present in the feces and intestines of mammals and has been associated with a range of inflammatory and metabolic disorders [58,59,60]. Research by Koji Hosomi found that the ingestion of *Blautia* wexlerae has a positive effect on obesity and type 2 diabetes through the restructuring of intestinal microbiota metabolism [61]. Moreover, SS showed a decreased abundance of the genus of *Blautia* compared to the healthy controls [62]. The genus *Butyricicoccus* and the genus *Subdoligranulum* are correlated with butyrate synthesis [33,63]. Studies have found that the genus *Butyricicoccus* appears to provide protection against IgA nephropathy and psychiatric disorders [64,65,66], which is consistent with our findings in SS. Similarly, the oral administration of *Butyricicoccus B pullicaecorum* has shown a significant protective effect in reducing intestinal myeloperoxidase, tumor necrosis factor α, and interleukin-12 levels in colitis. This supports its potential use as a pharmabiotic to maintain the integrity of epithelial tight junctions [67,68]. The genus *Escherichia.Shigella* can injure the colon epithelium, triggering strong inflammatory reactions that lead to the deterioration of the colon epithelium or affect amino acid metabolism, resulting in gut dysbiosis and a compromised intestinal barrier integrity. For example, a previous study reported that *Escherichia.Shigella* was significantly increased in various diseases, such as primary SS [69], rheumatoid arthritis [70], acute radiation-induced intestinal injury [71], Kashin–Beck disease [72], and IgA nephropathy [73], suggesting that it could be used as a microbial diagnostic biomarker or a target for therapy. In SLE patients with depression, the abundance of the genus *Subdoligranulum* was decreased compared to the healthy controls, and it showed a negative correlation with IL-2 and IL-6 at the genus level. This indicated that the dysregulation of *Subdoligranulum* may enhance the inflammatory response, which has been associated with depression in SLE patients [63]. As we all know, short-chain fatty acids (SCFAs) are crucial for maintaining intestinal homeostasis, and they have been identified as a factor that can impact lipid metabolism through their interaction with the composition of the gut microbiota. Studies have shown a strong positive correlation between the abundance of *Escherichia.Shigella* and plasma lipid levels, as well as a negative correlation between the genus *Subdoligranulum* and plasma lipid levels, through correlation analysis [74,75]. A more comprehensive grasp of the interaction between the gut microbiota and autoimmune diseases or inflammation related diseases may become a new candidate diagnostic biomarker and an encouraging target for therapy.

In recent years, the dysbiosis of the gut microbiota has also been associated with SS, with diversity levels significantly lower than those in the healthy controls, which correlate with overall disease severity [76]. Correlation analyses demonstrated that opportunistic pathogens (such as *Bacteroides*, *Megamonas*, and *Veillonella)* showed positive associations with clinical characteristics in primary SS patients. Conversely, some probiotic genera (including *Collinsella*, unidentified_*Ruminococcaceae*, *Romboutsia*, and *Dorea*) exhibited negative correlations [77]. Furthermore, recent studies have reported that certain drugs may be effective in treating SS and are considered as potential treatment strategies for SS. For example, rapamycin, known as the strongest mTOR inhibitor, has been proven to reduce B cell proliferation and IgG production in patients with SS [78]. Metformin is known for its anti-inflammatory and immunomodulatory properties [79], and Kim, J.W. et al. have reported that metformin’s effects include decreasing the populations of Th17 and Th1 cells, inducing regulatory T cells, regulating B cell differentiation, and ameliorating salivary gland inflammation in SS animal models [80]. The short-chain fatty acid butyrate might alleviate SS by boosting the production of IL-10-producing B (B10) cells and reducing the number of IL-17-producing B cells [81]. Moreover, supplementation with propionate has been shown to regulate the Th17/Treg balance, while *L. acidophilus* may improve SS by boosting immunomodulation through the SINGR3 pathway [28]. A clinical trial has reported that a combination of probiotics, including *Lactobacillus bulgaricus*, *Lactobacillus acidophilus*, *Bifidobacterium bifidum*, and *Streptococcus thermophilus*, significantly down-regulated the candidial load from baseline to the end of the treatment in SS patients with oral candidiasis [82]. Hence, modulating the gut microbiome could offer an effective targeted approach for treating SS.

The advantages of our work as follows. Firstly, previous observational studies have failed to establish the temporal sequence between gut microbiota colonization and the pathological process of SS, as fecal samples were only collected after the onset. In contrast, the MR analysis is a suitable approach for examining causal associations because it removes potential confounders, reverse causation, and errors from non-differentially measured exposures. Secondly, the genetic variants of the gut microbiota utilized in our research were obtained from the most comprehensive available GWAS meta-analysis, ensuring the accuracy and dependability of the tools employed in the MR analysis. Thirdly, a number of horizontal pleiotropy analyses, such as the MR-Egger regression and MR-PRESSO, were conducted to ensure the consistency of causal estimates and validate the reliability of our outcomes.

There are also some limitations that must be mentioned in our study. First, the exposure factors only offered data at the taxonomic level of genus, which hindered our ability to investigate the causal connection between the gut microbiota and SS at the species level. When analyzing the connection between the gut microbiota and SS, it is crucial to take into account demographic variables such as age, sex, BMI, eating habits, and medication. However, the datasets available from the MiBioGen and FinnGen consortia do not provide sufficient information for subgroup analyses based on these characteristics. In addition, to include more genetic variations as instrumental variables for the sensitivity analysis and horizontal pleiotropy detection, we included SNPs in the analysis that met the locus-wide significance level, which had a lower GWAS significance threshold (*p* < 1 × 10^–5^). SS cases were identified using the ICD-10 code M35.0, which corresponds specifically to SS, as per international diagnostic standards. The FinnGen consortium leverages high-quality national health registry data, and diagnoses are made by clinicians in Finland, who likely follow international criteria, such as the 2016 ACR/EULAR classification, for SS. While our dataset does not differentiate between primary and secondary SS, we acknowledge this as a limitation and discuss the potential for overlap with other autoimmune conditions in our study. Finally, since most participants in the GWAS meta-analysis of gut microbiota data were of European descent, stratified analyses may not be applicable. Further research with more diverse populations and comprehensive demographic data is necessary to clarify these potential variations.

## 5. Conclusions

In summary, we have found a causal relationship between gut microbiota and SS. These strains of microorganisms have the potential to be used as new indicators and provide a wide range of possible targets for addressing or avoiding SS. Our two-step MR result calculated that the genus *Eubacterium coprostanoligenes* group has a protective effect by reducing CXCL6 levels in SS. Furthermore, the use of a more comprehensive method that integrates multiple omics platforms is urgently needed to improve our comprehension of SS pathogenesis, particularly in the context of intricate gene–environment interactions over time.

## Figures and Tables

**Table 1:** MR findings on the relationship between gut microbiome and the risk of SS.

Group	Gut Microbiota	MR Method	No. SNPs	OR (95% CI)	*p*-Value	F-Statistic
Phylum	*Lentisphaerae*	Inverse variance weighted	9	0.79 (0.63–0.99)	0.037	22.05
		MR-Egger	9	0.49 (0.21–1.11)	0.13	
		Weighted median	9	0.79 (0.59–1.06)	0.12	
		Simple mode	9	0.78 (0.51–1.19)	0.29	
		Weighted mode	9	0.79 (0.52–1.20)	0.30	
Class	*Deltaproteobacteria*	Inverse variance weighted	11	0.67 (0.47–0.96)	0.030	21.30
		MR-Egger	11	0.97 (0.15–6.40)	0.97	
		Weighted median	11	0.81 (0.50–1.33)	0.40	
		Simple mode	11	0.92 (0.41–2.09)	0.85	
		Weighted mode	11	0.93 (0.42–2.07)	0.86	
Family	*Porphyromonadaceae*	Inverse variance weighted	**9**	0.60 (0.38–0.94)	0.026	20.55
		MR-Egger	9	0.15 (0.02–1.12)	0.11	
		Weighted median	9	0.80 (0.45–1.43)	0.45	
		Simple mode	9	0.80 (0.31–2.05)	0.65	
		Weighted mode	9	0.85 (0.34–2.11)	0.73	
Genus	*Eubacterium coprostanoligenes* group	Inverse variance weighted	13	0.61 (0.40–0.93)	0.021	21.74
		MR-Egger	13	0.18 (0.04–0.83)	0.05	
		Weighted median	13	0.55 (0.32–0.93)	0.026	
		Simple mode	13	0.53 (0.22–1.28)	0.18	
		Weighted mode	13	0.52 (0.22–1.21)	0.16	

## Data Availability

Full data on the study can be obtained from the corresponding author.

## Supplementary Materials

The following supporting information can be downloaded at: https://www.mdpi.com/article/10.3390/biomedicines12102378/s1, Figure S1: Estimated proportion of the association between genus *Eubacterium coprostanoligenes* group and SS mediated by CXCL6 levels; Table S1: Instrumental variables used in MR analysis of the association between gut microbiota and SS; Table S2: Full MR results of causal links between gut microbiome and SS risk; Table S3: The heterogeneity of gut microbiota instrumental variables; Table S4: Directional horizontal pleiotropy assessed by intercept term in MR Egger regression of the association between gut microbiota and SS; Table S5: MR-PRESSO analysis for the association between gut microbiota and SS; Table S6: MR analysis of Sjogren’s syndrome in genus *Eubacterium coprostanoligenes* group and EBI; Table S7: MR analysis of 91 inflammatory factors and Sjogren’s syndrome; Table S8: MR analysis of genus Eubacterium coprostanoligenes group and 9 inflammatory factors.
