# Supplementary material for: Causal Relationship Between Sjögren’s Syndrome and Gut Microbiota: A Two-Sample Mendelian Randomization Study
Source: Biomedicines. 2024 Oct 18;12(10):2378. doi: 10.3390/biomedicines12102378 (PMC11505323; doi:10.3390/biomedicines12102378)
Supplement: Supplementary file 1 [file biomedicines-12-02378-s001.zip › Supplementary Table S6.pdf]

Table S6 MR analysis of Sjogren's syndrome in genus *Eubacterium coprostanoligenes* group and EBI.

Exposure	Outcome	Method	No.SNP	Beta	SE	OR (95% CI)	P-value
genus *Eubacterium coprostanoligenes* group	Sjogren's syndrome (ebi-a-GCST90013879)	Inverse variance weighted	13	-0.8966	0.40775	0.407953 (0.183454-0.90718)	0.027885
genus *Eubacterium coprostanoligenes* group	Sjogren's syndrome (ebi-a-GCST90013879)	MR Egger	13	0.684916	1.641997	1.983604 (0.079389-49.56227)	0.684613
genus *Eubacterium coprostanoligenes* group	Sjogren's syndrome (ebi-a-GCST90013879)	Weighted median	13	-0.758	0.539235	0.468604 (0.162854-1.348379)	0.159816
genus *Eubacterium coprostanoligenes* group	Sjogren's syndrome (ebi-a-GCST90013879)	Weighted mode	13	-0.47526	0.913804	0.621721 (0.103693-3.727698)	0.612458
