# Supplementary material for: Causal Relationship Between Sjögren’s Syndrome and Gut Microbiota: A Two-Sample Mendelian Randomization Study
Source: Biomedicines. 2024 Oct 18;12(10):2378. doi: 10.3390/biomedicines12102378 (PMC11505323; doi:10.3390/biomedicines12102378)
Supplement: Supplementary file 1 [file biomedicines-12-02378-s001.zip › Supplementary Table S7.pdf]

Table S7 MR analysis of 91 inflammatory factors and Sjogren's syndrome.

Exposure	Outcome	Method	No.snp	Beta	SE	95% CI	P-value
Adenosine Deaminase levels	Sjogren's syndrome	Inverse variance weighted	4	0.020006	0.125188	1.020207932 (0.798227084-1.303919955)	0.87303
Adenosine Deaminase levels	Sjogren's syndrome	MR Egger	4	0.041088	0.186675	1.041943848 (0.722674972-1.50226177)	0.846214
Adenosine Deaminase levels	Sjogren's syndrome	Weighted median	4	0.012811	0.130728	1.012893389 (0.783944777-1.308705725)	0.921935
Adenosine Deaminase levels	Sjogren's syndrome	Weighted mode	4	0.012197	0.135997	1.01227143 (0.775414054-1.321479077)	0.934191
Artemin levels	Sjogren's syndrome	Inverse variance weighted	25	0.16109	0.098856	1.17479035 (0.967860358-1.425962282)	0.103198
Artemin levels	Sjogren's syndrome	MR Egger	25	-0.04717	0.20538	0.953929276 (0.637813238-1.426720251)	0.820393
Artemin levels	Sjogren's syndrome	Weighted median	25	0.129449	0.139802	1.138200594 (0.865399305-1.496997495)	0.354478
Artemin levels	Sjogren's syndrome	Weighted mode	25	0.080013	0.242432	1.083301259 (0.673576728-1.742253807)	0.744231
Axin-1 levels	Sjogren's syndrome	Inverse variance weighted	12	0.135653	0.151995	1.145284148 (0.850221748-1.542745503)	0.372136
Axin-1 levels	Sjogren's syndrome	MR Egger	12	-0.20408	0.39189	0.815398924 (0.378257064-1.757734271)	0.613873

Axin-1 levels	Sjogren's syndrome	Weighted median	12	0.079787	0.206861	1.083056602 (0.722050334-1.624556555)	0.699716
Axin-1 levels	Sjogren's syndrome	Weighted mode	12	0.103121	0.315926	1.108625774 (0.596847188-2.059239165)	0.750241
beta-nerve growth factor levels	Sjogren's syndrome	Inverse variance weighted	29	0.116628	0.115371	1.123701368 (0.896283289-1.40882328)	0.312066
beta-nerve growth factor levels	Sjogren's syndrome	MR Egger	29	0.403862	0.28221	1.497597505 (0.861337508-2.603855362)	0.163883
beta-nerve growth factor levels	Sjogren's syndrome	Weighted median	29	0.116932	0.15732	1.124043253 (0.825789448-1.530018625)	0.457315
beta-nerve growth factor levels	Sjogren's syndrome	Weighted mode	29	-0.171	0.234109	0.842819758 (0.532668629-1.333559188)	0.47119
Caspase 8 levels	Sjogren's syndrome	Inverse variance weighted	17	0.122304	0.150159	1.130097366 (0.841973277-1.516817803)	0.41536
Caspase 8 levels	Sjogren's syndrome	MR Egger	17	-0.15808	0.28517	0.853778354 (0.488206575-1.4930923)	0.587511
Caspase 8 levels	Sjogren's syndrome	Weighted median	17	0.038508	0.192657	1.03925893 (0.712411885-1.516059946)	0.841576
Caspase 8 levels	Sjogren's syndrome	Weighted mode	17	-0.00608	0.228475	0.993940703 (0.635153381-1.555400868)	0.979107
C-C motif chemokine 19 levels	Sjogren's syndrome	Inverse variance weighted	25	-0.17785	0.116064	0.837065046 (0.66675095-1.050883979)	0.12543
C-C motif	Sjogren's syndrome	MR Egger	25	-0.43648	0.219162	0.646307782 (0.420615479-	0.058421

chemokine 19 levels						0.993101231)	
C-C motif chemokine 19 levels	Sjogren's syndrome	Weighted median	25	-0.37282	0.15504	0.688791829 (0.508294505-0.933384444)	0.016188
C-C motif chemokine 19 levels	Sjogren's syndrome	Weighted mode	25	-0.38717	0.161796	0.67897235 (0.494456802-0.932343229)	0.024889
C-C motif chemokine 20 levels	Sjogren's syndrome	Inverse variance weighted	26	0.155147	0.105798	1.16783008 (0.949123673-1.436932967)	0.142525
C-C motif chemokine 20 levels	Sjogren's syndrome	MR Egger	26	0.188385	0.286151	1.207298121 (0.689030139-2.11539187)	0.516585
C-C motif chemokine 20 levels	Sjogren's syndrome	Weighted median	26	0.13126	0.144949	1.140264523 (0.858267538-1.514915949)	0.365167
C-C motif chemokine 20 levels	Sjogren's syndrome	Weighted mode	26	-0.00951	0.283328	0.990532256 (0.568453765-1.726005191)	0.973482
C-C motif chemokine 23 levels	Sjogren's syndrome	Inverse variance weighted	27	0.060153	0.078794	1.061998553 (0.910024421-1.239352373)	0.445216
C-C motif chemokine 23 levels	Sjogren's syndrome	MR Egger	27	0.031448	0.113604	1.031947771 (0.825954842-1.289315284)	0.784192

C-C motif chemokine 23 levels	Sjogren's syndrome	Weighted median	27	0.021945	0.089324	1.022187951 (0.858018736-1.217768522)	0.805927
C-C motif chemokine 23 levels	Sjogren's syndrome	Weighted mode	27	0.010073	0.088142	1.010123857 (0.849858932-1.200611263)	0.909893
C-C motif chemokine 25 levels	Sjogren's syndrome	Inverse variance weighted	14	-0.00716	0.046699	0.992862942 (0.906021331-1.08802827)	0.878099
C-C motif chemokine 25 levels	Sjogren's syndrome	MR Egger	14	-0.06665	0.068514	0.935526833 (0.817966591-1.069983133)	0.349892
C-C motif chemokine 25 levels	Sjogren's syndrome	Weighted median	14	-0.04859	0.059789	0.952568288 (0.847233304-1.07099938)	0.416359
C-C motif chemokine 25 levels	Sjogren's syndrome	Weighted mode	14	-0.02732	0.055565	0.973048645 (0.872642989-1.085006901)	0.631131
C-C motif chemokine 28 levels	Sjogren's syndrome	Inverse variance weighted	30	0.097397	0.111966	1.102297984 (0.88509798-1.372798122)	0.384367
C-C motif chemokine 28 levels	Sjogren's syndrome	MR Egger	30	0.310403	0.239169	1.363974285 (0.85353477-2.17967201)	0.204934
C-C motif chemokine 28	Sjogren's syndrome	Weighted median	30	-0.01803	0.151213	0.982133039 (0.730222813-1.320946552)	0.905096

levels							
C-C motif chemokine 28 levels	Sjogren's syndrome	Weighted mode	30	-0.18114	0.329971	0.834315638 (0.436972058-1.592968178)	0.587229
C-C motif chemokine 4 levels	Sjogren's syndrome	Inverse variance weighted	25	-0.02726	0.05552	0.97310811 (0.872773207-1.084977616)	0.62343
C-C motif chemokine 4 levels	Sjogren's syndrome	MR Egger	25	0.0159	0.076007	1.016027557 (0.875400911-1.179244827)	0.836136
C-C motif chemokine 4 levels	Sjogren's syndrome	Weighted median	25	-0.01101	0.071015	0.989054164 (0.860540057-1.136760726)	0.876834
C-C motif chemokine 4 levels	Sjogren's syndrome	Weighted mode	25	-0.01128	0.064944	0.988781808 (0.870600268-1.123006161)	0.863549
CD40L receptor levels	Sjogren's syndrome	Inverse variance weighted	21	-0.16643	0.070795	0.846678056 (0.736980828-0.972703364)	0.018726
CD40L receptor levels	Sjogren's syndrome	MR Egger	21	-0.23336	0.09702	0.791868544 (0.654738846-0.957718935)	0.026512
CD40L receptor levels	Sjogren's syndrome	Weighted median	21	-0.20716	0.073743	0.812887427 (0.703491656-0.939294679)	0.004966
CD40L receptor levels	Sjogren's syndrome	Weighted mode	21	-0.21	0.077897	0.810582597 (0.695808655-0.94428855)	0.013903
CUB domain-	Sjogren's syndrome	Inverse	25	0.144816	0.109218	1.155826459 (0.933091475-	0.184863

containing protein 1 levels		variance weighted (multiplicative random effects)				1.431729729)	
CUB domain-containing protein 1 levels	Sjogren's syndrome	MR Egger	25	0.33726	0.209479	1.401103249 (0.929304991-2.112428463)	0.121039
CUB domain-containing protein 1 levels	Sjogren's syndrome	Weighted median	25	0.256406	0.127403	1.292277114 (1.006719411-1.658833753)	0.04416
CUB domain-containing protein 1 levels	Sjogren's syndrome	Weighted mode	25	0.236706	0.136616	1.267068792 (0.969416034-1.656113852)	0.095994
C-X-C motif chemokine 1 levels	Sjogren's syndrome	Inverse variance weighted	20	0.063633	0.107614	1.065700974 (0.863043103-1.315946518)	0.554316
C-X-C motif chemokine 1 levels	Sjogren's syndrome	MR Egger	20	0.081111	0.173355	1.084491678 (0.772081767-1.523313011)	0.645478
C-X-C motif chemokine 1 levels	Sjogren's syndrome	Weighted median	20	0.072008	0.120748	1.074664293 (0.848184556-1.361617983)	0.550939
C-X-C motif chemokine 1 levels	Sjogren's syndrome	Weighted mode	20	0.078023	0.115115	1.081147083 (0.862774523-1.354790834)	0.50608

C-X-C motif chemokine 10 levels	Sjogren's syndrome	Inverse variance weighted (multiplicative random effects)	26	-0.16107	0.135232	0.851233625 (0.653036055-1.109584499)	0.233631
C-X-C motif chemokine 10 levels	Sjogren's syndrome	MR Egger	26	-0.2391	0.25023	0.787336916 (0.482126436-1.285761108)	0.348842
C-X-C motif chemokine 10 levels	Sjogren's syndrome	Weighted median	26	-0.04964	0.14352	0.951576123 (0.718251623-1.260696237)	0.72946
C-X-C motif chemokine 10 levels	Sjogren's syndrome	Weighted mode	26	-0.08888	0.169583	0.914958314 (0.656220264-1.275712991)	0.604831
C-X-C motif chemokine 11 levels	Sjogren's syndrome	Inverse variance weighted	33	0.21877	0.088118	1.244545411 (1.047136584-1.479170246)	0.013039
C-X-C motif chemokine 11 levels	Sjogren's syndrome	MR Egger	33	0.221848	0.221609	1.248381473 (0.808555543-1.927457323)	0.32454
C-X-C motif chemokine 11 levels	Sjogren's syndrome	Weighted median	33	0.153369	0.128426	1.165754802 (0.906335562-1.499427272)	0.232391
C-X-C motif chemokine 11	Sjogren's syndrome	Weighted mode	33	0.115154	0.15101	1.122046418 (0.834581286-1.508526715)	0.45131

levels							
C-X-C motif chemokine 5 levels	Sjogren's syndrome	Inverse variance weighted	17	-0.04278	0.078486	0.958123978 (0.821509831-1.117456569)	0.585727
C-X-C motif chemokine 5 levels	Sjogren's syndrome	MR Egger	17	0.033628	0.115008	1.034199779 (0.825481316-1.29569157)	0.77399
C-X-C motif chemokine 5 levels	Sjogren's syndrome	Weighted median	17	-0.03999	0.09191	0.960797241 (0.802410747-1.150447377)	0.663474
C-X-C motif chemokine 5 levels	Sjogren's syndrome	Weighted mode	17	-0.02846	0.098423	0.97194012 (0.801420346-1.178741719)	0.776163
C-X-C motif chemokine 6 levels	Sjogren's syndrome	Inverse variance weighted	5	0.409613	0.187557	1.506235284 (1.042896438-2.175426676)	0.028967
C-X-C motif chemokine 6 levels	Sjogren's syndrome	MR Egger	5	0.297183	0.370668	1.346060979 (0.650947232-2.783451667)	0.481326
C-X-C motif chemokine 6 levels	Sjogren's syndrome	Weighted median	5	0.350853	0.221809	1.420278667 (0.919529332-2.193721746)	0.1137
C-X-C motif chemokine 6 levels	Sjogren's syndrome	Weighted mode	5	0.306849	0.249499	1.359136345 (0.833460453-2.216363834)	0.286138
C-X-C motif	Sjogren's syndrome	Inverse	27	-0.07358	0.102631	0.929059512 (0.759769505-	0.4734

chemokine 9 levels		variance weighted				1.136070309)	
C-X-C motif chemokine 9 levels	Sjogren's syndrome	MR Egger	27	-0.43894	0.218279	0.644721832 (0.420309848-0.988951941)	0.055238
C-X-C motif chemokine 9 levels	Sjogren's syndrome	Weighted median	27	-0.11904	0.15158	0.887771551 (0.659590029-1.194891207)	0.432257
C-X-C motif chemokine 9 levels	Sjogren's syndrome	Weighted mode	27	-0.1309	0.246866	0.877301508 (0.540770005-1.423262992)	0.600427
Cystatin D levels	Sjogren's syndrome	Inverse variance weighted	14	-0.28374	0.124125	0.752964469 (0.590360264-0.960355101)	0.02226
Cystatin D levels	Sjogren's syndrome	MR Egger	14	-0.05875	0.220606	0.942937833 (0.611926974-1.453003048)	0.794503
Cystatin D levels	Sjogren's syndrome	Weighted median	14	-0.07306	0.145027	0.929542294 (0.699551944-1.235146131)	0.614409
Cystatin D levels	Sjogren's syndrome	Weighted mode	14	-0.10642	0.138419	0.899046192 (0.685420484-1.179252844)	0.455736
Delta and Notch-like epidermal growth factor-related receptor levels	Sjogren's syndrome	Inverse variance weighted	20	0.072319	0.127271	1.074998268 (0.837669879-1.379566468)	0.569878
Delta and Notch-	Sjogren's syndrome	MR Egger	20	-0.36535	0.303735	0.69395055 (0.382634531-	0.244609

like epidermal growth factor-related receptor levels						1.258556997)	
Delta and Notch-like epidermal growth factor-related receptor levels	Sjogren's syndrome	Weighted median	20	0.063836	0.165166	1.065917526 (0.771137418-1.473382236)	0.699129
Delta and Notch-like epidermal growth factor-related receptor levels	Sjogren's syndrome	Weighted mode	20	0.134587	0.200209	1.144064569 (0.772732699-1.69383765)	0.509527
Eotaxin levels	Sjogren's syndrome	Inverse variance weighted	24	-0.01396	0.102324	0.986132832 (0.80692982-1.205133258)	0.891449
Eotaxin levels	Sjogren's syndrome	MR Egger	24	-0.15846	0.185922	0.853457052 (0.592818788-1.228687338)	0.403234
Eotaxin levels	Sjogren's syndrome	Weighted median	24	-0.07358	0.137312	0.929065201 (0.709845381-1.215986144)	0.592074
Eotaxin levels	Sjogren's syndrome	Weighted mode	24	-0.07697	0.134401	0.925921555 (0.711492227-1.204975533)	0.572431
Eukaryotic translation initiation factor	Sjogren's syndrome	Inverse variance weighted	15	0.016258	0.134135	1.01639061 (0.781416521-1.322022051)	0.903529

4E-binding protein 1 levels							
Eukaryotic translation initiation factor 4E-binding protein 1 levels	Sjogren's syndrome	MR Egger	15	0.058538	0.361528	1.060284794 (0.522015823-2.153581934)	0.873861
Eukaryotic translation initiation factor 4E-binding protein 1 levels	Sjogren's syndrome	Weighted median	15	-0.02916	0.168669	0.971261345 (0.697849595-1.351793577)	0.862745
Eukaryotic translation initiation factor 4E-binding protein 1 levels	Sjogren's syndrome	Weighted mode	15	-0.11456	0.205577	0.891755059 (0.596011966-1.334246845)	0.586137
Fibroblast growth factor 19 levels	Sjogren's syndrome	Inverse variance weighted	28	-0.10981	0.092187	0.896003406 (0.747890631-1.073448537)	0.233586
Fibroblast growth factor 19 levels	Sjogren's syndrome	MR Egger	28	-0.01892	0.218789	0.981262665 (0.639069901-1.506684034)	0.931768
Fibroblast growth factor 19 levels	Sjogren's syndrome	Weighted median	28	-0.02231	0.134633	0.977932979 (0.751115826-1.273242926)	0.868361
Fibroblast growth factor 19 levels	Sjogren's syndrome	Weighted mode	28	0.025225	0.161595	1.025545857 (0.747141602-1.407690728)	0.877115

Fibroblast growth factor 21 levels	Sjogren's syndrome	Inverse variance weighted	24	0.059903	0.103469	1.061733025 (0.86684347-1.300438955)	0.562629
Fibroblast growth factor 21 levels	Sjogren's syndrome	MR Egger	24	0.110182	0.238301	1.116481582 (0.699851092-1.781137641)	0.648359
Fibroblast growth factor 21 levels	Sjogren's syndrome	Weighted median	24	-0.04942	0.144322	0.951782972 (0.717280235-1.262952444)	0.732037
Fibroblast growth factor 21 levels	Sjogren's syndrome	Weighted mode	24	-0.10187	0.224776	0.903146779 (0.58133281-1.403110388)	0.654647
Fibroblast growth factor 23 levels	Sjogren's syndrome	Inverse variance weighted (multiplicative random effects)	19	-0.03494	0.173645	0.965665126 (0.687095577-1.357175283)	0.840538
Fibroblast growth factor 23 levels	Sjogren's syndrome	MR Egger	19	-0.117	0.402342	0.889584316 (0.404303097-1.957344035)	0.774722
Fibroblast growth factor 23 levels	Sjogren's syndrome	Weighted median	19	0.245039	0.197614	1.277671209 (0.86737384-1.882053208)	0.214981
Fibroblast growth factor 23 levels	Sjogren's syndrome	Weighted mode	19	0.2927	0.335396	1.340041289 (0.694421187-2.585909948)	0.39432
Fibroblast growth factor 5 levels	Sjogren's syndrome	Inverse variance weighted	7	0.058404	0.070368	1.060143157 (0.923561522-1.216923278)	0.406553
Fibroblast growth factor 5 levels	Sjogren's syndrome	MR Egger	7	-0.08826	0.098464	0.915518317 (0.754836876-1.110403871)	0.411106

Fibroblast growth factor 5 levels	Sjogren's syndrome	Weighted median	7	0.033476	0.06393	1.034043045 (0.912263577-1.172079042)	0.600528
Fibroblast growth factor 5 levels	Sjogren's syndrome	Weighted mode	7	0.030936	0.063468	1.031419281 (0.910773786-1.168046061)	0.643252
Fms-related tyrosine kinase 3 ligand levels	Sjogren's syndrome	Inverse variance weighted (multiplicative random effects)	37	0.009643	0.096577	1.009689909 (0.835563969-1.220102529)	0.920463
Fms-related tyrosine kinase 3 ligand levels	Sjogren's syndrome	MR Egger	37	0.076895	0.165299	1.079928222 (0.781068572-1.493140303)	0.644681
Fms-related tyrosine kinase 3 ligand levels	Sjogren's syndrome	Weighted median	37	0.16344	0.118986	1.177554146 (0.932605488-1.486838524)	0.169565
Fms-related tyrosine kinase 3 ligand levels	Sjogren's syndrome	Weighted mode	37	0.157069	0.134035	1.170076528 (0.899749121-1.521623137)	0.248954
Fractalkine levels	Sjogren's syndrome	Inverse variance weighted	24	0.066132	0.11868	1.068368001 (0.846638761-1.348166701)	0.57737
Fractalkine levels	Sjogren's syndrome	MR Egger	24	0.372982	0.320946	1.452058357 (0.774086623-2.723821095)	0.257634
Fractalkine levels	Sjogren's syndrome	Weighted median	24	0.286444	0.155611	1.331683015 (0.981616566-1.806590998)	0.065656

Fractalkine levels	Sjogren's syndrome	Weighted mode	24	0.341927	0.236636	1.407657222 (0.885254705-2.238337559)	0.16196
Glial cell line-derived neurotrophic factor levels	Sjogren's syndrome	Inverse variance weighted	17	0.000817	0.108106	1.000817555 (0.809716524-1.2370203)	0.993969
Glial cell line-derived neurotrophic factor levels	Sjogren's syndrome	MR Egger	17	-0.05349	0.182075	0.947919606 (0.663416257-1.354431052)	0.77297
Glial cell line-derived neurotrophic factor levels	Sjogren's syndrome	Weighted median	17	-0.02928	0.141716	0.971145358 (0.735619981-1.282079513)	0.836318
Glial cell line-derived neurotrophic factor levels	Sjogren's syndrome	Weighted mode	17	-0.01266	0.132951	0.987421566 (0.760908998-1.281363937)	0.92533
Hepatocyte growth factor levels	Sjogren's syndrome	Inverse variance weighted	27	0.006564	0.110749	1.00658556 (0.810175071-1.250611782)	0.952738
Hepatocyte growth factor levels	Sjogren's syndrome	MR Egger	27	0.023219	0.242083	1.023490412 (0.63682251-1.644936551)	0.924355
Hepatocyte growth factor	Sjogren's syndrome	Weighted median	27	0.05468	0.156949	1.05620222(0.776513438-1.436630809)	0.727547

levels							
Hepatocyte growth factor levels	Sjogren's syndrome	Weighted mode	27	0.158173	0.229963	1.171369116 (0.746354999-1.83840881)	0.497656
Interferon gamma levels	Sjogren's syndrome	Inverse variance weighted	17	-0.0539	0.115798	0.947531507 (0.755134181-1.188948903)	0.641629
Interferon gamma levels	Sjogren's syndrome	MR Egger	17	-0.21753	0.211631	0.804506963 (0.531356756-1.218073256)	0.320308
Interferon gamma levels	Sjogren's syndrome	Weighted median	17	-0.15835	0.162031	0.853553362 (0.621308275-1.172611682)	0.328438
Interferon gamma levels	Sjogren's syndrome	Weighted mode	17	-0.32411	0.241759	0.723171719 (0.450248192-1.161531229)	0.198767
Interleukin-10 levels	Sjogren's syndrome	Inverse variance weighted	27	-0.11776	0.10462	0.888909536 (0.724107299-1.091219718)	0.260338
Interleukin-10 levels	Sjogren's syndrome	MR Egger	27	-0.26647	0.211144	0.766081896 (0.506460755-1.158789632)	0.218592
Interleukin-10 levels	Sjogren's syndrome	Weighted median	27	-0.19112	0.142115	0.826037392 (0.62521394-1.091366858)	0.178693
Interleukin-10 levels	Sjogren's syndrome	Weighted mode	27	-0.22427	0.161485	0.799100285 (0.582294016-1.096630309)	0.17668
Interleukin-10 receptor subunit alpha levels	Sjogren's syndrome	Inverse variance weighted	18	0.04721	0.10344	1.048342303 (0.855959868-1.283963917)	0.648101
Interleukin-10	Sjogren's syndrome	MR Egger	18	-0.07621	0.184714	0.926626128 (0.645168305-	0.685406

receptor subunit alpha levels						1.330871299)	
Interleukin-10 receptor subunit alpha levels	Sjogren's syndrome	Weighted median	18	-0.02823	0.154082	0.972163161 (0.718756076-1.314912308)	0.854622
Interleukin-10 receptor subunit alpha levels	Sjogren's syndrome	Weighted mode	18	-0.18929	0.188983	0.827544511 (0.57138133-1.198551443)	0.330563
Interleukin-10 receptor subunit beta levels	Sjogren's syndrome	Inverse variance weighted (multiplicative random effects)	24	0.118614	0.082158	1.125935282 (0.958470734-1.322659331)	0.148816
Interleukin-10 receptor subunit beta levels	Sjogren's syndrome	MR Egger	24	0.034018	0.12118	1.034603249 (0.815874215-1.311971704)	0.781546
Interleukin-10 receptor subunit beta levels	Sjogren's syndrome	Weighted median	24	0.045264	0.074175	1.046304161 (0.904729747-1.2100325)	0.541707
Interleukin-10 receptor subunit beta levels	Sjogren's syndrome	Weighted mode	24	0.058792	0.072294	1.060554603 (0.920439278-1.221999205)	0.424422
Interleukin-12 subunit beta levels	Sjogren's syndrome	Inverse variance weighted	16	0.02416	0.068931	1.024453934 (0.894987418-1.172648735)	0.725971

Interleukin-12 subunit beta levels	Sjogren's syndrome	MR Egger	16	-0.03412	0.103846	0.966456295 (0.788473538-1.18461524)	0.747353
Interleukin-12 subunit beta levels	Sjogren's syndrome	Weighted median	16	-0.03782	0.066676	0.962885044 (0.844926241-1.097311888)	0.570551
Interleukin-12 subunit beta levels	Sjogren's syndrome	Weighted mode	16	-0.02442	0.065566	0.975874643 (0.858189316-1.109698408)	0.714756
Interleukin-13 levels	Sjogren's syndrome	Inverse variance weighted	22	0.19937	0.093492	1.220633473 (1.016255602-1.466113518)	0.032968
Interleukin-13 levels	Sjogren's syndrome	MR Egger	22	0.255545	0.18008	1.291164883 (0.90718161-1.837676973)	0.171279
Interleukin-13 levels	Sjogren's syndrome	Weighted median	22	0.172946	0.138509	1.18880144 (0.906166981-1.559589892)	0.211802
Interleukin-13 levels	Sjogren's syndrome	Weighted mode	22	0.286671	0.187759	1.331986045 (0.921883385-1.924524137)	0.141732
Interleukin-15 receptor subunit alpha levels	Sjogren's syndrome	Inverse variance weighted	18	0.07369	0.080504	1.076472704 (0.919341647-1.26046012)	0.360004
Interleukin-15 receptor subunit alpha levels	Sjogren's syndrome	MR Egger	18	-0.06494	0.129638	0.937128292 (0.726856387-1.208229646)	0.623266
Interleukin-15 receptor subunit	Sjogren's syndrome	Weighted median	18	-0.02489	0.082832	0.975416371 (0.829244196-1.147354545)	0.763796

alpha levels							
Interleukin-15 receptor subunit alpha levels	Sjogren's syndrome	Weighted mode	18	0.004338	0.081158	1.004347199 (0.856645109-1.177515969)	0.957998
Interleukin-17A levels	Sjogren's syndrome	Inverse variance weighted	17	0.181156	0.120791	1.198601965 (0.945923159-1.518777353)	0.133679
Interleukin-17A levels	Sjogren's syndrome	MR Egger	17	0.257077	0.288521	1.293144642 (0.734603224-2.276362274)	0.38699
Interleukin-17A levels	Sjogren's syndrome	Weighted median	17	0.103726	0.169132	1.109296126 (0.79630498-1.545309807)	0.53969
Interleukin-17A levels	Sjogren's syndrome	Weighted mode	17	0.133814	0.247455	1.143180075 (0.703843844-1.856748048)	0.596122
Interleukin-17C levels	Sjogren's syndrome	Inverse variance weighted	27	0.122524	0.109694	1.130346552 (0.911670654-1.401474669)	0.264011
Interleukin-17C levels	Sjogren's syndrome	MR Egger	27	0.002538	0.285658	1.002541312 (0.572723773-1.754928171)	0.992981
Interleukin-17C levels	Sjogren's syndrome	Weighted median	27	0.131951	0.148386	1.141052446 (0.853094798-1.52620868)	0.373872
Interleukin-17C levels	Sjogren's syndrome	Weighted mode	27	0.210527	0.249967	1.234328679 (0.75623167-2.014683262)	0.407345
Interleukin-18 levels	Sjogren's syndrome	Inverse variance weighted	24	-0.15784	0.085374	0.85398978 (0.722405322-1.009542043)	0.064492
Interleukin-18	Sjogren's syndrome	MR Egger	24	0.061668	0.179383	1.063608971 (0.748321572-	0.734277

levels						1.511735175)	
Interleukin-18 levels	Sjogren's syndrome	Weighted median	24	-0.06761	0.123321	0.934627927 (0.73394923-1.190176822)	0.583542
Interleukin-18 levels	Sjogren's syndrome	Weighted mode	24	-0.0352	0.128503	0.965412745 (0.750462407-1.241929988)	0.786591
interleukin-18 receptor 1 levels	Sjogren's syndrome	Inverse variance weighted	10	0.123716	0.085518	1.131694748 (0.957051318-1.338207241)	0.147988
interleukin-18 receptor 1 levels	Sjogren's syndrome	MR Egger	10	0.130073	0.213897	1.138911724 (0.748889052-1.732058856)	0.559977
interleukin-18 receptor 1 levels	Sjogren's syndrome	Weighted median	10	0.107574	0.09884	1.113573804 (0.917455058-1.351615653)	0.276431
interleukin-18 receptor 1 levels	Sjogren's syndrome	Weighted mode	10	0.109396	0.088061	1.115604154 (0.938751593-1.325774186)	0.245531
Interleukin-1-alpha levels	Sjogren's syndrome	Inverse variance weighted	18	-0.13239	0.122588	0.875995282 (0.688895004-1.113911015)	0.280143
Interleukin-1-alpha levels	Sjogren's syndrome	MR Egger	18	-0.17731	0.252584	0.837517 (0.510493023-1.374033914)	0.492766
Interleukin-1-alpha levels	Sjogren's syndrome	Weighted median	18	-0.19959	0.172022	0.819069828 (0.584646099-1.147489711)	0.245952
Interleukin-1-alpha levels	Sjogren's syndrome	Weighted mode	18	-0.46905	0.224544	0.625598111 (0.402865242-0.971473725)	0.052067
Interleukin-2 levels	Sjogren's syndrome	Inverse variance weighted	17	-0.34	0.16494	0.711767821 (0.515155522-0.983418424)	0.039267

		(multiplicative random effects)					
Interleukin-2 levels	Sjogren's syndrome	MR Egger	17	0.012146	0.444681	1.012219832 (0.423403787-2.419886215)	0.97857
Interleukin-2 levels	Sjogren's syndrome	Weighted median	17	-0.21306	0.187902	0.808104037 (0.559141909-1.167918419)	0.256831
Interleukin-2 levels	Sjogren's syndrome	Weighted mode	17	-0.15293	0.27021	0.858192828 (0.505333052-1.457444602)	0.579276
Interleukin-2 receptor subunit beta levels	Sjogren's syndrome	Inverse variance weighted	19	0.06291	0.127934	1.064930469 (0.828746833-1.368423816)	0.622907
Interleukin-2 receptor subunit beta levels	Sjogren's syndrome	MR Egger	19	-0.02573	0.281465	0.974595275 (0.561353183-1.692046963)	0.928223
Interleukin-2 receptor subunit beta levels	Sjogren's syndrome	Weighted median	19	0.009282	0.171732	1.009324758 (0.720858029-1.413227605)	0.956898
Interleukin-2 receptor subunit beta levels	Sjogren's syndrome	Weighted mode	19	-0.03603	0.292108	0.96461228 (0.544133014-1.710017269)	0.903204
Interleukin-20 levels	Sjogren's syndrome	Inverse variance weighted	22	0.197988	0.130344	1.218947339 (0.94413433-1.573751285)	0.128772
Interleukin-20 levels	Sjogren's syndrome	MR Egger	22	0.81698	0.263717	2.263652394 (1.349986057-3.795685245)	0.005671

Interleukin-20 levels	Sjogren's syndrome	Weighted median	22	0.051186	0.160208	1.052519037 (0.768878842-1.440794389)	0.749348
Interleukin-20 levels	Sjogren's syndrome	Weighted mode	22	0.048375	0.236645	1.04956455 (0.660044059-1.668957897)	0.839992
Interleukin-20 receptor subunit alpha levels	Sjogren's syndrome	Inverse variance weighted	14	0.020672	0.130509	1.020887057 (0.790470779-1.318467943)	0.874146
Interleukin-20 receptor subunit alpha levels	Sjogren's syndrome	MR Egger	14	0.046141	0.248536	1.047221593 (0.643398742-1.70449986)	0.855821
Interleukin-20 receptor subunit alpha levels	Sjogren's syndrome	Weighted median	14	-0.02293	0.182312	0.977331923 (0.683683726-1.397104613)	0.899916
Interleukin-20 receptor subunit alpha levels	Sjogren's syndrome	Weighted mode	14	-0.05833	0.235474	0.943337886 (0.594604079-1.496603199)	0.808223
Interleukin-22 receptor subunit alpha-1 levels	Sjogren's syndrome	Inverse variance weighted	17	0.088898	0.144139	1.092969368 (0.823975907-1.449777874)	0.537397
Interleukin-22 receptor subunit alpha-1 levels	Sjogren's syndrome	MR Egger	17	0.382454	0.27392	1.465877524 (0.856903674-2.50762948)	0.182968
Interleukin-22 receptor subunit alpha-1 levels	Sjogren's syndrome	Weighted median	17	-0.06595	0.201429	0.93618168 (0.630812471-1.389376684)	0.743373
Interleukin-22	Sjogren's syndrome	Weighted	17	-0.13415	0.311349	0.874461515 (0.475023896-	0.67232

receptor subunit alpha-1 levels		mode				1.609777843)	
Interleukin-24 levels	Sjogren's syndrome	Inverse variance weighted	15	-0.10944	0.150944	0.896334428 (0.666781665-1.204915265)	0.468423
Interleukin-24 levels	Sjogren's syndrome	MR Egger	15	-0.29445	0.379251	0.744938155 (0.354238079-1.566553363)	0.451403
Interleukin-24 levels	Sjogren's syndrome	Weighted median	15	0.033474	0.217407	1.03404103 (0.675269497-1.58342833)	0.877632
Interleukin-24 levels	Sjogren's syndrome	Weighted mode	15	0.129589	0.317375	1.138360854 (0.611117985-2.120483223)	0.689218
Interleukin-33 levels	Sjogren's syndrome	Inverse variance weighted	18	0.066657	0.133172	1.068929298 (0.823361787-1.387737277)	0.616697
Interleukin-33 levels	Sjogren's syndrome	MR Egger	18	0.177325	0.303306	1.194019211 (0.65891886-2.163668342)	0.566943
Interleukin-33 levels	Sjogren's syndrome	Weighted median	18	-0.03643	0.175147	0.964221265 (0.684051794-1.35914072)	0.835211
Interleukin-33 levels	Sjogren's syndrome	Weighted mode	18	-0.21865	0.265022	0.803599001 (0.478021906-1.350924187)	0.420774
Interleukin-4 levels	Sjogren's syndrome	Inverse variance weighted (multiplicative random effects)	19	-0.23409	0.1511	0.791287057 (0.588457073-1.064028686)	0.121318

Interleukin-4 levels	Sjogren's syndrome	MR Egger	19	0.119789	0.365423	1.127259504 (0.55076929-2.307162025)	0.747058
Interleukin-4 levels	Sjogren's syndrome	Weighted median	19	-0.28621	0.184956	0.751107808 (0.522714708-1.079294177)	0.12176
Interleukin-4 levels	Sjogren's syndrome	Weighted mode	19	-0.40806	0.371988	0.664941493 (0.320730906-1.378561217)	0.287118
Interleukin-5 levels	Sjogren's syndrome	Inverse variance weighted (multiplicative random effects)	21	-0.04669	0.134879	0.954384331 (0.732675626-1.243182412)	0.729228
Interleukin-5 levels	Sjogren's syndrome	MR Egger	21	-0.18994	0.280226	0.827010369 (0.477504508-1.432334436)	0.506068
Interleukin-5 levels	Sjogren's syndrome	Weighted median	21	0.061905	0.172424	1.063860968 (0.758777621-1.491609833)	0.719576
Interleukin-5 levels	Sjogren's syndrome	Weighted mode	21	0.174396	0.227408	1.190526451 (0.762369842-1.859141264)	0.452108
Interleukin-6 levels	Sjogren's syndrome	Inverse variance weighted	12	-0.03601	0.132008	0.964634314 (0.744724325-1.249481625)	0.785039
Interleukin-6 levels	Sjogren's syndrome	MR Egger	12	-0.11404	0.257536	0.892220068 (0.538583261-1.478056797)	0.66732
Interleukin-6 levels	Sjogren's syndrome	Weighted median	12	-0.01085	0.171145	0.989206836 (0.707302806-1.383467103)	0.949442
Interleukin-6	Sjogren's syndrome	Weighted	12	-0.00037	0.180687	0.999629137 (0.701511526-	0.998399

levels		mode				1.424436198)	
Interleukin-7 levels	Sjogren's syndrome	Inverse variance weighted	21	-0.00406	0.157784	0.995944898 (0.731016556-1.356886149)	0.979455
Interleukin-7 levels	Sjogren's syndrome	MR Egger	21	0.028283	0.468171	1.028687261 (0.410930174-2.57512723)	0.952458
Interleukin-7 levels	Sjogren's syndrome	Weighted median	21	-0.03488	0.198217	0.965724771 (0.654829103-1.424225541)	0.860332
Interleukin-7 levels	Sjogren's syndrome	Weighted mode	21	-0.23408	0.367975	0.791296792 (0.384691716-1.627668565)	0.531901
Interleukin-8 levels	Sjogren's syndrome	Inverse variance weighted	26	0.017095	0.113104	1.017242355 (0.814983243-1.269697283)	0.879859
Interleukin-8 levels	Sjogren's syndrome	MR Egger	26	-0.21535	0.197768	0.806261922 (0.547183375-1.188008109)	0.287017
Interleukin-8 levels	Sjogren's syndrome	Weighted median	26	0.10936	0.165021	1.115564178 (0.807283125-1.541569985)	0.507519
Interleukin-8 levels	Sjogren's syndrome	Weighted mode	26	0.267669	0.277357	1.306914096 (0.758850733-2.250804249)	0.343753
Latency-associated peptide transforming growth factor beta 1 levels	Sjogren's syndrome	Inverse variance weighted	28	0.072661	0.090582	1.07536568 (0.900431861-1.284285237)	0.422466
Latency-associated peptide	Sjogren's syndrome	MR Egger	28	0.129826	0.1618	1.138630439 (0.829193258-1.563542955)	0.429603

transforming growth factor beta 1 levels							
Latency-associated peptide transforming growth factor beta 1 levels	Sjogren's syndrome	Weighted median	28	-0.02332	0.139926	0.97695018 (0.742617197-1.285226977)	0.86764
Latency-associated peptide transforming growth factor beta 1 levels	Sjogren's syndrome	Weighted mode	28	-0.0429	0.142598	0.958005676 (0.724412689-1.266922693)	0.765829
Leukemia inhibitory factor levels	Sjogren's syndrome	Inverse variance weighted	20	-0.03766	0.119939	0.963042301 (0.761291788-1.218258869)	0.75354
Leukemia inhibitory factor levels	Sjogren's syndrome	MR Egger	20	0.164363	0.284943	1.178641868 (0.674270027-2.060297205)	0.5712
Leukemia inhibitory factor levels	Sjogren's syndrome	Weighted median	20	0.087935	0.170379	1.091916706 (0.781915217-1.524822726)	0.605777
Leukemia inhibitory factor levels	Sjogren's syndrome	Weighted mode	20	0.223969	0.278233	1.251032321 (0.725156325-2.158268244)	0.430796
Leukemia	Sjogren's syndrome	Inverse	22	0.036179	0.092922	1.036841409 (0.864202699-	0.697018

inhibitory factor receptor levels		variance weighted				1.243967544)	
Leukemia inhibitory factor receptor levels	Sjogren's syndrome	MR Egger	22	0.311077	0.157019	1.36489456 (1.003325099-1.856763237)	0.061493
Leukemia inhibitory factor receptor levels	Sjogren's syndrome	Weighted median	22	0.109051	0.12789	1.115219492 (0.867956232-1.432923077)	0.39383
Leukemia inhibitory factor receptor levels	Sjogren's syndrome	Weighted mode	22	0.132993	0.132472	1.142242136 (0.881040074-1.480882806)	0.326839
Macrophage colony-stimulating factor 1 levels	Sjogren's syndrome	Inverse variance weighted	21	0.006989	0.104728	1.007013361 (0.82014215-1.236463594)	0.946794
Macrophage colony-stimulating factor 1 levels	Sjogren's syndrome	MR Egger	21	0.099294	0.237346	1.104391086 (0.693569333-1.758554787)	0.680381
Macrophage colony-stimulating factor 1 levels	Sjogren's syndrome	Weighted median	21	0.154887	0.155793	1.167525761 (0.860305504-1.584456215)	0.320134
Macrophage colony-stimulating factor	Sjogren's syndrome	Weighted mode	21	0.148876	0.184067	1.160528639 (0.809049372-1.664702761)	0.42814

1 levels							
Macrophage inflammatory protein 1a levels	Sjogren's syndrome	Inverse variance weighted (multiplicative random effects)	18	-0.07715	0.107243	0.925750327 (0.750251584-1.142301711)	0.471893
Macrophage inflammatory protein 1a levels	Sjogren's syndrome	MR Egger	18	0.054577	0.170808	1.056094061 (0.755627442-1.476037798)	0.753464
Macrophage inflammatory protein 1a levels	Sjogren's syndrome	Weighted median	18	-0.03081	0.096499	0.969655367 (0.802557086-1.171544737)	0.74948
Macrophage inflammatory protein 1a levels	Sjogren's syndrome	Weighted mode	18	-0.04083	0.091755	0.959996465 (0.801984762-1.149140553)	0.661975
Matrix metalloproteinase-1 levels	Sjogren's syndrome	Inverse variance weighted	22	-0.09714	0.095227	0.907428622 (0.752927836-1.09363297)	0.307686
Matrix metalloproteinase-1 levels	Sjogren's syndrome	MR Egger	22	-0.23161	0.173546	0.793253905 (0.564529733-1.11464768)	0.197008
Matrix metalloproteinase-1 levels	Sjogren's syndrome	Weighted median	22	-0.15884	0.123191	0.853133668 (0.670122756-1.086124966)	0.197271
Matrix	Sjogren's syndrome	Weighted	22	-0.16215	0.155219	0.850317747 (0.627271906-	0.308075

metalloproteinase-1 levels		mode				1.152674406)	
Matrix metalloproteinase-10 levels	Sjogren's syndrome	Inverse variance weighted	20	0.141359	0.089914	1.151837686 (0.965728175-1.373813138)	0.115915
Matrix metalloproteinase-10 levels	Sjogren's syndrome	MR Egger	20	0.256995	0.138899	1.293038056 (0.984867842-1.697636316)	0.080767
Matrix metalloproteinase-10 levels	Sjogren's syndrome	Weighted median	20	0.164518	0.121252	1.178825009 (0.92947548-1.495067306)	0.174834
Matrix metalloproteinase-10 levels	Sjogren's syndrome	Weighted mode	20	0.221689	0.124577	1.248183165 (0.977769727-1.593382543)	0.091153
Monocyte chemoattractant protein 2 levels	Sjogren's syndrome	Inverse variance weighted (multiplicative random effects)	5	0.301939	0.272125	1.352478374 (0.793400803-2.305515378)	0.26719
Monocyte chemoattractant protein 2 levels	Sjogren's syndrome	MR Egger	5	0.774417	0.800376	2.16932734 (0.451888191-10.4140387)	0.404632
Monocyte chemoattractant protein 2 levels	Sjogren's syndrome	Weighted median	5	0.483228	0.184227	1.621299152 (1.129915703-2.326377919)	0.008716

Monocyte chemoattractant protein 2 levels	Sjogren's syndrome	Weighted mode	5	0.544595	0.195971	1.723910819 (1.174089667-2.531210858)	0.049871
Monocyte chemoattractant protein-1 levels	Sjogren's syndrome	Inverse variance weighted	23	-0.00601	0.073867	0.994011421 (0.860032218-1.148862431)	0.93519
Monocyte chemoattractant protein-1 levels	Sjogren's syndrome	MR Egger	23	-0.09149	0.124417	0.91257031 (0.715089027-1.16458866)	0.470261
Monocyte chemoattractant protein-1 levels	Sjogren's syndrome	Weighted median	23	-0.03251	0.10615	0.968009209 (0.786180956-1.191890775)	0.759377
Monocyte chemoattractant protein-1 levels	Sjogren's syndrome	Weighted mode	23	-0.05265	0.11486	0.948712875 (0.757466997-1.188244667)	0.651179
Monocyte chemoattractant protein-3 levels	Sjogren's syndrome	Inverse variance weighted	23	-0.23593	0.092471	0.789837121 (0.658908108-0.946782518)	0.01073
Monocyte chemoattractant protein-3 levels	Sjogren's syndrome	MR Egger	23	0.012166	0.197421	1.012240058 (0.687441127-1.490498452)	0.951445
Monocyte chemoattractant protein-3 levels	Sjogren's syndrome	Weighted median	23	-0.25252	0.139295	0.776839814 (0.591235923-1.020709454)	0.069856
Monocyte chemoattractant	Sjogren's syndrome	Weighted mode	23	-0.24788	0.149746	0.780450582 (0.581941247-1.046674581)	0.112048

protein-3 levels							
Monocyte chemoattractant protein-4 levels	Sjogren's syndrome	Inverse variance weighted	24	0.129112	0.074292	1.137817777 (0.983635093-1.316168264)	0.082228
Monocyte chemoattractant protein-4 levels	Sjogren's syndrome	MR Egger	24	0.032747	0.147055	1.033289385 (0.774544759-1.37847031)	0.825833
Monocyte chemoattractant protein-4 levels	Sjogren's syndrome	Weighted median	24	0.09524	0.107721	1.099922545 (0.890569767-1.35848942)	0.376625
Monocyte chemoattractant protein-4 levels	Sjogren's syndrome	Weighted mode	24	0.089887	0.109236	1.094050474 (0.883189689-1.355254092)	0.419032
Natural killer cell receptor 2B4 levels	Sjogren's syndrome	Inverse variance weighted	28	0.044783	0.079716	1.045800894 (0.894526129-1.22265798)	0.574267
Natural killer cell receptor 2B4 levels	Sjogren's syndrome	MR Egger	28	0.102881	0.154693	1.108359104 (0.818470867-1.500920746)	0.511867
Natural killer cell receptor 2B4 levels	Sjogren's syndrome	Weighted median	28	0.121279	0.125235	1.12893956 (0.88321891-1.443022241)	0.332841
Natural killer cell receptor 2B4 levels	Sjogren's syndrome	Weighted mode	28	0.14903	0.137482	1.160707738 (0.886534118-1.519673553)	0.287953
Neurotrophin-3	Sjogren's syndrome	Inverse	23	-0.00866	0.110286	0.991376792 (0.798658555-	0.937408

levels		variance weighted				1.230598404)	
Neurotrophin-3 levels	Sjogren's syndrome	MR Egger	23	-0.24369	0.221808	0.783734101 (0.507413851-1.210528921)	0.284364
Neurotrophin-3 levels	Sjogren's syndrome	Weighted median	23	-0.17031	0.157724	0.843404996 (0.61912515-1.148930853)	0.280239
Neurotrophin-3 levels	Sjogren's syndrome	Weighted mode	23	-0.20835	0.223776	0.811918922 (0.523637874-1.258908816)	0.361914
Neurturin levels	Sjogren's syndrome	Inverse variance weighted	19	-0.00554	0.100229	0.994475501 (0.817104363-1.210349091)	0.955922
Neurturin levels	Sjogren's syndrome	MR Egger	19	0.142117	0.177716	1.152711192 (0.813664621-1.633035355)	0.434927
Neurturin levels	Sjogren's syndrome	Weighted median	19	-0.01584	0.142573	0.984285687 (0.744320971-1.301613619)	0.911542
Neurturin levels	Sjogren's syndrome	Weighted mode	19	-0.05494	0.220171	0.94653787 (0.614787222-1.457307353)	0.805759
Oncostatin-M levels	Sjogren's syndrome	Inverse variance weighted	20	-0.10033	0.120263	0.90453628 (0.714587908-1.144975829)	0.404126
Oncostatin-M levels	Sjogren's syndrome	MR Egger	20	-0.00096	0.26354	0.999043076 (0.596011401-1.674610694)	0.997141
Oncostatin-M levels	Sjogren's syndrome	Weighted median	20	0.003349	0.142833	1.00335501 (0.758354835-1.327506899)	0.981292
Oncostatin-M levels	Sjogren's syndrome	Weighted mode	20	0.041332	0.181709	1.042197639 (0.729921184-1.48807288)	0.822496

Osteoprotegerin levels	Sjogren's syndrome	Inverse variance weighted	21	0.067859	0.122632	1.070214812 (0.841558869-1.360997768)	0.580018
Osteoprotegerin levels	Sjogren's syndrome	MR Egger	21	-0.19123	0.25788	0.825945994 (0.498241516-1.369188965)	0.467439
Osteoprotegerin levels	Sjogren's syndrome	Weighted median	21	0.052763	0.134003	1.054180187 (0.810679519-1.370820207)	0.693767
Osteoprotegerin levels	Sjogren's syndrome	Weighted mode	21	0.02847	0.136542	1.028878913 (0.787295398-1.344592919)	0.836943
Programmed cell death 1 ligand 1 levels	Sjogren's syndrome	Inverse variance weighted	22	-0.11317	0.132111	0.893000815 (0.689281593-1.156929858)	0.39166
Programmed cell death 1 ligand 1 levels	Sjogren's syndrome	MR Egger	22	-0.28681	0.305845	0.750650762 (0.412190295-1.367030165)	0.359545
Programmed cell death 1 ligand 1 levels	Sjogren's syndrome	Weighted median	22	-0.0208	0.176931	0.979419638 (0.692408132-1.385400868)	0.906438
Programmed cell death 1 ligand 1 levels	Sjogren's syndrome	Weighted mode	22	0.191019	0.247539	1.210483044 (0.745159223-1.966384036)	0.448905
Protein S100-A12 levels	Sjogren's syndrome	Inverse variance weighted	23	0.021	0.105938	1.021221615 (0.829743284-1.256887047)	0.842869
Protein S100-A12 levels	Sjogren's syndrome	MR Egger	23	0.242415	0.208203	1.274323342 (0.847331566-1.916487057)	0.257352

Protein S100-A12 levels	Sjogren's syndrome	Weighted median	23	0.059782	0.13779	1.061604809 (0.810352238-1.390759126)	0.664389
Protein S100-A12 levels	Sjogren's syndrome	Weighted mode	23	0.081388	0.171258	1.084792096(0.775477095-1.517483753)	0.639301
Signaling lymphocytic activation molecule levels	Sjogren's syndrome	Inverse variance weighted	31	0.005405	0.093466	1.005420031 (0.837120461-1.207555526)	0.953882
Signaling lymphocytic activation molecule levels	Sjogren's syndrome	MR Egger	31	0.131529	0.216884	1.140571373 (0.745602306-1.744768017)	0.548934
Signaling lymphocytic activation molecule levels	Sjogren's syndrome	Weighted median	31	0.033047	0.132893	1.033598777 (0.796583102-1.341136198)	0.803615
Signaling lymphocytic activation molecule levels	Sjogren's syndrome	Weighted mode	31	0.120743	0.218777	1.128335362 (0.734871729-1.73246655)	0.585104
SIR2-like protein 2 levels	Sjogren's syndrome	Inverse variance weighted	18	0.083084	0.121178	1.086633223 (0.856908218-1.377944261)	0.492941
SIR2-like protein 2 levels	Sjogren's syndrome	MR Egger	18	-0.27172	0.226383	0.762068861 (0.488982039-1.187669286)	0.247509
SIR2-like protein	Sjogren's syndrome	Weighted	18	0.117224	0.166244	1.124370855 (0.811707884-	0.48073

2 levels		median				1.557468942)	
SIR2-like protein 2 levels	Sjogren's syndrome	Weighted mode	18	0.109517	0.310169	1.115739009 (0.607493301-2.049197141)	0.728363
STAM binding protein levels	Sjogren's syndrome	Inverse variance weighted	18	0.29462	0.159637	1.342615641 (0.981897341-1.835850536)	0.064956
STAM binding protein levels	Sjogren's syndrome	MR Egger	18	0.401631	0.402092	1.494259156 (0.679451509-3.286195405)	0.332734
STAM binding protein levels	Sjogren's syndrome	Weighted median	18	0.106394	0.208286	1.112260461 (0.739451504-1.673028354)	0.609485
STAM binding protein levels	Sjogren's syndrome	Weighted mode	18	-0.02548	0.321515	0.974837011 (0.519102679-1.830672881)	0.937747
Stem cell factor levels	Sjogren's syndrome	Inverse variance weighted (multiplicative random effects)	41	0.05972	0.078436	1.061538982 (0.910269285-1.237946867)	0.446429
Stem cell factor levels	Sjogren's syndrome	MR Egger	41	0.089572	0.140074	1.093705682 (0.831126057-1.439242711)	0.526263
Stem cell factor levels	Sjogren's syndrome	Weighted median	41	0.083519	0.104511	1.087106091 (0.885747978-1.334239175)	0.424209
Stem cell factor levels	Sjogren's syndrome	Weighted mode	41	0.142819	0.103821	1.153520619 (0.941134209-1.413836418)	0.176591
Sulfotransferase 1A1 levels	Sjogren's syndrome	Inverse variance	27	-0.11087	0.085108	0.895058182 (0.757539809-1.05754066)	0.192694

		weighted					
Sulfotransferase 1A1 levels	Sjogren's syndrome	MR Egger	27	-0.1316	0.206304	0.876693663 (0.585111454-1.313581837)	0.52935
Sulfotransferase 1A1 levels	Sjogren's syndrome	Weighted median	27	-0.21442	0.1291	0.807006889 (0.626592179-1.039368413)	0.096733
Sulfotransferase 1A1 levels	Sjogren's syndrome	Weighted mode	27	-0.20104	0.163178	0.817883441 (0.594006769-1.126137542)	0.228975
T-cell surface glycoprotein CD5 levels	Sjogren's syndrome	Inverse variance weighted	21	-0.00891	0.104151	0.99113076 (0.808119468-1.21558782)	0.931834
T-cell surface glycoprotein CD5 levels	Sjogren's syndrome	MR Egger	21	0.056168	0.235842	1.057774975 (0.666255174-1.679368417)	0.814308
T-cell surface glycoprotein CD5 levels	Sjogren's syndrome	Weighted median	21	0.14251	0.148544	1.153164149 (0.861882854-1.542886658)	0.337368
T-cell surface glycoprotein CD5 levels	Sjogren's syndrome	Weighted mode	21	0.104625	0.184124	1.110294563 (0.773942535-1.592823707)	0.576202
T-cell surface glycoprotein CD6 isoform levels	Sjogren's syndrome	Inverse variance weighted	5	0.146661	0.261039	1.157961729 (0.694213358-1.931503262)	0.574228
T-cell surface glycoprotein CD6 isoform levels	Sjogren's syndrome	MR Egger	5	1.203695	1.004468	3.332408017 (0.46530537-23.86592528)	0.316819
T-cell surface	Sjogren's syndrome	Weighted	5	0.314041	0.207414	1.368945612 (0.91165794-	0.130006

glycoprotein CD6 isoform levels		median				2.055608806)	
T-cell surface glycoprotein CD6 isoform levels	Sjogren's syndrome	Weighted mode	5	0.319844	0.224914	1.376912429 (0.8860455-2.139718372)	0.22807
Thymic stromal lymphopoietin levels	Sjogren's syndrome	Inverse variance weighted (multiplicative random effects)	20	0.105241	0.156595	1.110978467 (0.817351993-1.510087655)	0.501546
Thymic stromal lymphopoietin levels	Sjogren's syndrome	MR Egger	20	0.158928	0.356376	1.172252983 (0.58299885-2.357083646)	0.660948
Thymic stromal lymphopoietin levels	Sjogren's syndrome	Weighted median	20	0.241737	0.169425	1.273458932 (0.913624167-1.775016152)	0.153635
Thymic stromal lymphopoietin levels	Sjogren's syndrome	Weighted mode	20	0.24629	0.226349	1.279270072 (0.820899429-1.993583941)	0.290163
TNF-beta levels	Sjogren's syndrome	Inverse variance weighted	6	0.146971	0.078477	1.158320596 (0.993180406-1.350919325)	0.061096
TNF-beta levels	Sjogren's syndrome	MR Egger	6	0.245113	0.125277	1.277765389 (0.999569372-1.633387772)	0.122035
TNF-beta levels	Sjogren's syndrome	Weighted	6	0.186998	0.071335	1.205625319 (1.048312819-	0.008757

		median				1.386544535)	
TNF-beta levels	Sjogren's syndrome	Weighted mode	6	0.200959	0.071675	1.222574154 (1.062342686-1.406973081)	0.037825
TNF-related activation-induced cytokine levels	Sjogren's syndrome	Inverse variance weighted	38	0.031755	0.068256	1.032264858 (0.903005499-1.180026853)	0.641761
TNF-related activation-induced cytokine levels	Sjogren's syndrome	MR Egger	38	0.052711	0.134747	1.054125276 (0.80945527-1.372750465)	0.697966
TNF-related activation-induced cytokine levels	Sjogren's syndrome	Weighted median	38	0.052699	0.097718	1.054112651 (0.870377036-1.276634648)	0.589681
TNF-related activation-induced cytokine levels	Sjogren's syndrome	Weighted mode	38	0.065728	0.120948	1.067935841 (0.842542974-1.353624676)	0.590088
TNF-related apoptosis-inducing ligand levels	Sjogren's syndrome	Inverse variance weighted	27	-0.03558	0.067518	0.965048189 (0.845428199-1.101593262)	0.598241
TNF-related apoptosis-inducing ligand levels	Sjogren's syndrome	MR Egger	27	-0.0355	0.106205	0.965126649 (0.783756397-1.188468064)	0.741
TNF-related apoptosis-inducing ligand	Sjogren's syndrome	Weighted median	27	-0.05531	0.08466	0.946194233 (0.801523668-1.116977031)	0.51357

levels							
TNF-related apoptosis-inducing ligand levels	Sjogren's syndrome	Weighted mode	27	-0.06032	0.072967	0.94146634 (0.816006514-1.086215435)	0.415972
Transforming growth factor-alpha levels	Sjogren's syndrome	Inverse variance weighted	15	-0.03997	0.14055	0.960816926 (0.72946142-1.265548993)	0.776109
Transforming growth factor-alpha levels	Sjogren's syndrome	MR Egger	15	-0.28826	0.298264	0.749563172 (0.41775444-1.344916763)	0.351443
Transforming growth factor-alpha levels	Sjogren's syndrome	Weighted median	15	-0.10941	0.19617	0.896360865 (0.610238068-1.31663828)	0.577021
Transforming growth factor-alpha levels	Sjogren's syndrome	Weighted mode	15	-0.16386	0.28284	0.848857399 (0.487614583-1.477722178)	0.571556
Tumor necrosis factor levels	Sjogren's syndrome	Inverse variance weighted	23	-0.27386	0.114277	0.760436808 (0.607839143-0.951344028)	0.016553
Tumor necrosis factor levels	Sjogren's syndrome	MR Egger	23	-0.20569	0.267688	0.814084103 (0.4817357-1.37571894)	0.450804
Tumor necrosis factor levels	Sjogren's syndrome	Weighted median	23	-0.33918	0.156594	0.712351719 (0.524082141-0.968254651)	0.03031
Tumor necrosis factor levels	Sjogren's syndrome	Weighted mode	23	-0.3312	0.229953	0.718059425 (0.457531006-1.126938571)	0.163861

Tumor necrosis factor ligand superfamily member 12 levels	Sjogren's syndrome	Inverse variance weighted	31	0.096388	0.094269	1.10118626 (0.915414265-1.324658382)	0.306553
Tumor necrosis factor ligand superfamily member 12 levels	Sjogren's syndrome	MR Egger	31	0.172147	0.207377	1.187852323 (0.791115271-1.783549368)	0.413255
Tumor necrosis factor ligand superfamily member 12 levels	Sjogren's syndrome	Weighted median	31	-0.02126	0.136627	0.978960818 (0.748972994-1.279571214)	0.876322
Tumor necrosis factor ligand superfamily member 12 levels	Sjogren's syndrome	Weighted mode	31	-0.05523	0.226703	0.946270619 (0.606794262-1.475669993)	0.809193
Tumor necrosis factor ligand superfamily member 14 levels	Sjogren's syndrome	Inverse variance weighted	33	-0.15172	0.075043	0.859232321 (0.741707965-0.995378528)	0.043205
Tumor necrosis factor ligand superfamily member 14 levels	Sjogren's syndrome	MR Egger	33	-0.2647	0.121215	0.767438823 (0.605150917-0.973248705)	0.036675
Tumor necrosis factor ligand	Sjogren's syndrome	Weighted median	33	-0.17881	0.116386	0.836268664 (0.665696281-1.050547071)	0.124461

superfamily member 14 levels							
Tumor necrosis factor ligand superfamily member 14 levels	Sjogren's syndrome	Weighted mode	33	-0.18682	0.118035	0.829594827 (0.658252229-1.045537784)	0.123318
Tumor necrosis factor receptor superfamily member 9 levels	Sjogren's syndrome	Inverse variance weighted (multiplicative random effects)	25	0.123951	0.120101	1.131959892 (0.894538021-1.432396575)	0.302048
Tumor necrosis factor receptor superfamily member 9 levels	Sjogren's syndrome	MR Egger	25	0.258553	0.273494	1.295054867 (0.757679135-2.213558523)	0.354297
Tumor necrosis factor receptor superfamily member 9 levels	Sjogren's syndrome	Weighted median	25	0.133162	0.143513	1.142435602 (0.862325396-1.513534347)	0.35347
Tumor necrosis factor receptor superfamily member 9 levels	Sjogren's syndrome	Weighted mode	25	0.193786	0.228255	1.213836093 (0.776007069-1.898691545)	0.404277
Urokinase-type plasminogen	Sjogren's syndrome	Inverse variance	33	0.139932	0.092461	1.150195858 (0.959549509-1.37872043)	0.130173

activator levels		weighted					
Urokinase-type plasminogen activator levels	Sjogren's syndrome	MR Egger	33	0.002434	0.186432	1.002436784 (0.69560552-1.444611172)	0.989668
Urokinase-type plasminogen activator levels	Sjogren's syndrome	Weighted median	33	0.060431	0.128092	1.062294379 (0.826438146-1.36546135)	0.637086
Urokinase-type plasminogen activator levels	Sjogren's syndrome	Weighted mode	33	0.031507	0.155493	1.032008244 (0.76089526-1.399720923)	0.840712
Vascular endothelial growth factor A levels	Sjogren's syndrome	Inverse variance weighted	24	-0.09759	0.067737	0.907019993 (0.794251343-1.035799656)	0.149661
Vascular endothelial growth factor A levels	Sjogren's syndrome	MR Egger	24	-0.16534	0.099557	0.847601233 (0.697343723-1.030234913)	0.110939
Vascular endothelial growth factor A levels	Sjogren's syndrome	Weighted median	24	-0.11926	0.07454	0.887579438 (0.766933264-1.027204447)	0.109618
Vascular endothelial growth factor A levels	Sjogren's syndrome	Weighted mode	24	-0.11929	0.075043	0.887547361 (0.766149037-1.028181567)	0.125565
