# Supplementary material for: Causal Relationship Between Sjögren’s Syndrome and Gut Microbiota: A Two-Sample Mendelian Randomization Study
Source: Biomedicines. 2024 Oct 18;12(10):2378. doi: 10.3390/biomedicines12102378 (PMC11505323; doi:10.3390/biomedicines12102378)
Supplement: Supplementary file 1 [file biomedicines-12-02378-s001.zip › Supplementary Table S8.pdf]

Table S8 MR analysis of genus *Eubacterium coprostanoligenes* group and 9 inflammatory factors.

Exposure	Outcome	Method	No. snp	Beta	SE	OR (95%CI)	P-value
genus *Eubacterium coprostanoligenes* group	CD40L receptor levels	Inverse variance weighted	11	-0.02446	0.064922	0.975836581 (0.85923903-1.108256257)	0.706352363
genus *Eubacterium coprostanoligenes* group	Cystatin D levels	Inverse variance weighted	11	-0.09861	0.069299	0.90609744 (0.791018768-1.03791794)	0.1547504
genus *Eubacterium coprostanoligenes* group	C-X-C motif chemokine 11 levels	Inverse variance weighted	11	0.121868	0.066846	1.129604882 (0.990890766-1.287737492)	0.068287587
genus *Eubacterium coprostanoligenes* group	C-X-C motif chemokine 6 levels	Inverse variance weighted	11	-0.14276	0.067053	0.866966215516059 (0.988732362492808-0.760196032359021)	0.033254681
genus *Eubacterium coprostanoligenes* group	Interleukin-13 levels	Inverse variance weighted	11	0.109854	0.076439	1.116115531 (0.960821916-1.296508601)	0.150675489
genus *Eubacterium coprostanoligenes* group	Interleukin-2 levels	Inverse variance weighted	11	0.036781	0.074657	1.037466037 (0.896239981-1.200945953)	0.622247424
genus *Eubacterium coprostanoligenes* group	Monocyte chemoattractant protein-3 levels	Inverse variance weighted	11	0.072828	0.095982	1.075545422 (0.891101842-1.298165821)	0.447991772
genus *Eubacterium*	Tumor necrosis	Inverse	11	-0.02197	0.085084	0.978271138	0.796254281

coprostanoligenes group	factor ligand superfamily member 14 levels	variance weighted				(0.828008068-1.155803253)	
genus *Eubacterium coprostanoligenes* group	Tumor necrosis factor levels	Inverse variance weighted (multiplicative random effects)	11	-0.01044	0.10358	0.989618437 (0.807790255-1.212374925)	0.919747908
genus *Eubacterium coprostanoligenes* group	CD40L receptor levels	MR Egger	11	-0.10211	0.165689	0.902932452 (0.652556245-1.249374317)	0.552988428
genus *Eubacterium coprostanoligenes* group	Cystatin D levels	MR Egger	11	0.117743	0.169431	1.124955188 (0.807072959-1.568041849)	0.504640908
genus *Eubacterium coprostanoligenes* group	C-X-C motif chemokine 11 levels	MR Egger	11	0.04403	0.171302	1.04501344 (0.746976722-1.461964019)	0.802934808
genus *Eubacterium coprostanoligenes* group	C-X-C motif chemokine 6 levels	MR Egger	11	0.100435	0.175302	1.105652107 (0.784148575-1.558973161)	0.580723771
genus *Eubacterium coprostanoligenes* group	Interleukin-13 levels	MR Egger	11	0.500962	0.19302	1.650308177 (1.130480107-2.409168515)	0.028956515
genus *Eubacterium*	Interleukin-2 levels	MR Egger	11	0.041356	0.191703	1.04222255	0.834010036

coprostanoligenes group						(0.715780584-1.51754304)	
genus *Eubacterium coprostanoligenes* group	Monocyte chemoattractant protein-3 levels	MR Egger	11	0.094018	0.260323	1.09857939 (0.659538968-1.829879256)	0.726312984
genus *Eubacterium coprostanoligenes* group	Tumor necrosis factor levels	MR Egger	11	0.506999	0.209193	1.660301323 (1.101839229-2.501817335)	0.038383666
genus *Eubacterium coprostanoligenes* group	Tumor necrosis factor ligand superfamily member 14 levels	MR Egger	11	-0.02906	0.231155	0.971356714 (0.617470135-1.528063972)	0.90271468
genus *Eubacterium coprostanoligenes* group	CD40L receptor levels	Weighted median	11	-0.03453	0.08437	0.96606101 (0.818817855-1.139782028)	0.6823576
genus *Eubacterium coprostanoligenes* group	Cystatin D levels	Weighted median	11	-0.07563	0.090195	0.927157561 (0.776922982-1.10644319)	0.401729963
genus *Eubacterium coprostanoligenes* group	C-X-C motif chemokine 11 levels	Weighted median	11	0.135983	0.084244	1.145662552 (0.971286074-1.351345106)	0.106491303
genus *Eubacterium coprostanoligenes* group	C-X-C motif chemokine 6 levels	Weighted median	11	0.183627	0.08988	1.201567476 (1.00749067-1.433030043)	0.04104942
genus Eubacterium coprostanoligenes	Interleukin-13 levels	Weighted median	11	0.079053	0.106794	1.08226117 (0.877864291-	0.459157181

group						1.334248645)	
genus *Eubacterium coprostanoligenes* group	Interleukin-2 levels	Weighted median	11	0.044427	0.096492	1.04542898 (0.865285016-1.263077174)	0.645210151
genus *Eubacterium coprostanoligenes* group	Monocyte chemoattractant protein-3 levels	Weighted median	11	0.077193	0.110539	1.080250831 (0.86982464-1.341582894)	0.484968605
genus *Eubacterium coprostanoligenes* group	Tumor necrosis factor levels	Weighted median	11	0.022393	0.109885	1.02264514 (0.824496809-1.268413743)	0.838524107
genus *Eubacterium coprostanoligenes* group	Tumor necrosis factor ligand superfamily member 14 levels	Weighted median	11	0.006608	0.105049	1.00663015 (0.81931344-1.236772412)	0.94984113
genus *Eubacterium coprostanoligenes* group	CD40L receptor levels	Weighted mode	11	-0.01597	0.119787	0.984160715 (0.778217578-1.244603489)	0.896610616
genus *Eubacterium coprostanoligenes* group	Cystatin D levels	Weighted mode	11	-0.03944	0.132616	0.961325722 (0.741286108-1.246680781)	0.772235762
genus *Eubacterium coprostanoligenes* group	C-X-C motif chemokine 11 levels	Weighted mode	11	0.127663	0.115172	1.136170442 (0.906581874-1.423901481)	0.293611221
genus *Eubacterium coprostanoligenes* group	C-X-C motif chemokine 6 levels	Weighted mode	11	0.210389	0.147947	1.234158334 (0.923497786-1.649323708)	0.185441613

genus *Eubacterium coprostanoligenes* group	Interleukin-13 levels	Weighted mode	11	0.065303	0.157541	1.067482292 (0.783896521-1.453659269)	0.687250551
genus *Eubacterium coprostanoligenes* group	Interleukin-2 levels	Weighted mode	11	0.030337	0.150862	1.030802303 (0.766936131-1.38545225)	0.844656284
genus *Eubacterium coprostanoligenes* group	Monocyte chemoattractant protein-3 levels	Weighted mode	11	0.052907	0.14459	1.054331631 (0.794144366-1.399764623)	0.722054317
genus *Eubacterium coprostanoligenes* group	Tumor necrosis factor levels	Weighted mode	11	0.0434	0.163369	1.044355701 (0.758203219-1.438504617)	0.795900154
genus *Eubacterium coprostanoligenes* group	Tumor necrosis factor ligand superfamily member 14 levels	Weighted mode	11	-0.01014	0.136876	0.989907354 (0.756978047-1.294511212)	0.94238416
