# Supplementary material for: Causal Relationship Between Sjögren’s Syndrome and Gut Microbiota: A Two-Sample Mendelian Randomization Study
Source: Biomedicines. 2024 Oct 18;12(10):2378. doi: 10.3390/biomedicines12102378 (PMC11505323; doi:10.3390/biomedicines12102378)
Supplement: Supplementary file 1 [file biomedicines-12-02378-s001.zip › Supplementary Tables S1-S5.pdf]

Table S1 Instrumental variables used in MR analysis of the association between gut microbiota and SS.

Group	Gut microbiota	SNP	Effect allele	Other allele	Exposure (Bacteria)			Outcome (Urolithiasis)		
					Beta	SE	P-value	Beta	SE	P-value
Phylum	Lentisphaerae	rs1002941	G	A	0.108	0.023	4.31E-06	0.063	0.037	0.093
		rs11770843	C	T	0.112	0.023	1.14E-06	-0.053	0.034	0.124
		rs17114848	G	A	0.149	0.032	6.77E-06	-0.056	0.054	0.303
		rs2031282	A	G	0.120	0.027	5.86E-06	-0.028	0.043	0.509
		rs2825714	A	G	-0.138	0.029	1.50E-06	0.018	0.043	0.681
		rs60995569	T	G	-0.161	0.034	9.19E-06	0.061	0.052	0.238
		rs62570196	C	T	-0.217	0.044	9.64E-07	0.025	0.081	0.755
		rs72640280	A	G	0.220	0.049	5.19E-06	-0.141	0.070	0.045
		rs77599476	A	G	0.230	0.048	1.90E-06	-0.050	0.072	0.487
Class	Deltaproteobacteria	rs1035691	A	G	-0.055	0.012	9.65172E-06	0.032	0.033	0.338
		rs11599763	T	C	-0.054	0.012	3.94123E-06	-0.021	0.033	0.530
		rs17084793	G	A	-0.071	0.016	5.68634E-06	0.014	0.046	0.761
		rs17791387	A	G	-0.074	0.015	1.60223E-06	0.091	0.055	0.098

		rs2838334	G	A	0.056	0.012	5.44551E-06	-0.080	0.034	0.017
		rs3935584	C	T	-0.052	0.012	7.50121E-06	0.005	0.032	0.877
		rs4506934	C	T	-0.094	0.020	3.58887E-06	0.020	0.050	0.684
		rs55744759	A	G	-0.078	0.017	7.3137E-06	0.069	0.051	0.172
		rs6058181	C	T	0.083	0.017	3.39554E-07	0.018	0.044	0.682
		rs62020470	A	G	-0.059	0.013	4.85084E-06	-0.005	0.042	0.914
		rs9928243	C	A	-0.054	0.012	5.02081E-06	0.045	0.032	0.163
Family	Porphyromonadaceae	rs10762312	G	A	-0.052	0.012	8.70354E-06	0.004	0.035	0.912
		rs10858364	G	T	0.055	0.012	4.31063E-06	-0.007	0.038	0.858
		rs17065783	A	G	-0.059	0.012	1.79351E-06	0.045	0.044	0.305
		rs1980561	A	G	-0.049	0.011	8.94542E-06	-0.015	0.032	0.637
		rs35233670	T	C	-0.047	0.011	7.90987E-06	0.027	0.032	0.402
		rs35961441	A	C	0.092	0.021	8.37393E-06	-0.169	0.083	0.042
		rs6953849	A	G	0.072	0.015	2.4448E-06	-0.007	0.042	0.858
		rs7330827	T	C	-0.104	0.024	8.04891E-06	0.116	0.068	0.090
		rs864093	A	C	-0.053	0.012	9.598E-06	0.066	0.039	0.093

Genus	Eubacterium coprostanoligenes group	rs1020520	T	G	-0.059	0.013	8.88975E-06	-0.038	0.045	0.401
		rs10444197	A	G	-0.051	0.011	5.98464E-06	-0.019	0.034	0.573
		rs11052069	T	C	0.048	0.011	9.37949E-06	-0.026	0.032	0.427
		rs11720857	C	T	0.063	0.014	9.26255E-06	-0.033	0.042	0.429
		rs12906958	C	T	-0.053	0.012	4.34803E-06	0.080	0.035	0.024
		rs17159861	C	T	0.096	0.017	1.03787E-08	-0.075	0.052	0.151
		rs2644213	G	A	0.054	0.012	9.85638E-06	-0.040	0.035	0.255
		rs4076415	T	G	0.052	0.011	1.98974E-06	0.047	0.033	0.156
		rs62024432	C	T	-0.077	0.017	7.50207E-06	0.092	0.054	0.092
		rs6762473	C	A	0.052	0.011	4.26113E-06	-0.009	0.034	0.787
		rs76898927	G	A	0.123	0.027	4.78979E-06	-0.092	0.071	0.195
		rs79895140	T	C	-0.064	0.014	8.61816E-06	0.000	0.051	0.995
		rs9648214	T	C	-0.083	0.016	2.5203E-07	0.142	0.057	0.013
Genus	Blautia	rs11149971	C	T	0.118	0.023	1.03934E-06	0.005	0.071	0.941
		rs115043014	G	A	-0.207	0.044	5.186E-06	0.193	0.125	0.124
		rs117001700	T	C	0.196	0.044	8.83912E-06	0.094	0.136	0.489

		rs12453000	C	T	0.063	0.013	1.26117E-06	-0.081	0.047	0.085
		rs16892041	T	C	-0.062	0.014	8.82028E-06	0.050	0.039	0.206
		rs2788271	T	G	-0.058	0.013	7.16388E-06	0.072	0.042	0.089
		rs3005511	A	G	0.050	0.011	6.18905E-06	-0.067	0.035	0.056
		rs4926264	T	C	0.083	0.018	5.10293E-06	0.008	0.052	0.876
		rs67794373	C	T	0.060	0.012	1.00003E-06	-0.023	0.038	0.549
		rs682885	A	G	-0.049	0.011	4.4908E-06	0.012	0.034	0.733
		rs72973581	A	G	0.125	0.027	1.73934E-06	-0.004	0.069	0.949
		rs7860714	A	G	-0.050	0.011	4.09147E-06	0.028	0.034	0.408
Genus	Butyricicoccus	rs10084203	A	G	0.055	0.012	8.58646E-06	-0.062	0.048	0.194
		rs12034718	A	G	0.070	0.016	9.57686E-06	-0.036	0.039	0.350
		rs12585793	T	C	-0.262	0.056	5.79191E-06	0.167	0.097	0.086
		rs2017189	G	T	-0.051	0.011	3.87256E-06	-0.005	0.032	0.875
		rs4962426	G	T	0.061	0.014	7.3849E-06	0.021	0.041	0.604
		rs56221232	T	C	0.083	0.017	7.6194E-07	-0.069	0.054	0.198
		rs62478070	T	G	0.224	0.049	5.9377E-06	-0.113	0.102	0.269

		rs7322368	T	C	0.082	0.018	5.5178E-06	-0.062	0.056	0.270
Genus	Escherichia.Shigella	rs112767262	T	C	0.073	0.016	8.20636E-06	-0.038	0.039	0.331
		rs113127095	A	G	0.151	0.032	3.33349E-06	-0.160	0.080	0.045
		rs113513883	A	G	0.172	0.038	5.27716E-06	0.051	0.092	0.579
		rs1154904	A	G	-0.061	0.013	3.03751E-06	0.062	0.032	0.053
		rs118526	C	A	-0.059	0.014	7.99566E-06	0.029	0.035	0.414
		rs2798105	A	G	-0.101	0.022	8.24501E-06	0.031	0.054	0.561
		rs4731451	G	A	-0.061	0.014	7.4731E-06	-0.025	0.035	0.460
		rs57024273	T	C	0.063	0.014	9.70464E-06	-0.051	0.037	0.162
		rs592299	T	C	-0.059	0.013	4.76927E-06	0.005	0.032	0.880
		rs73208162	A	G	-0.119	0.025	2.19391E-06	-0.055	0.091	0.548
Genus	Subdoligranulum	rs10065321	T	C	-0.051	0.011	2.10155E-06	0.018	0.033	0.584
		rs10497836	C	T	-0.052	0.012	8.37954E-06	0.030	0.039	0.445
		rs1667315	G	A	0.049	0.011	6.72143E-06	-0.071	0.033	0.030
		rs2114677	C	T	-0.104	0.023	2.71726E-06	0.090	0.050	0.072
		rs2171249	C	T	0.107	0.023	4.50556E-06	-0.080	0.062	0.200

rs35940633	G	A	-0.051	0.011	4.21698E-06	0.006	0.034	0.858
rs3761728	T	G	-0.054	0.012	3.86747E-06	-0.014	0.037	0.707
rs4347804	A	G	0.166	0.036	2.18405E-06	-0.092	0.091	0.315
rs6555306	T	C	-0.074	0.016	2.80664E-06	0.045	0.046	0.330
rs75158211	T	C	-0.072	0.016	7.52162E-06	0.038	0.045	0.402
rs76528319	G	T	-0.143	0.031	7.41394E-06	0.012	0.058	0.836

Abbreviations: MR, Mendelian randomization study; SS, Sjögren's syndrome; SNP, single nucleotide polymorphism.

Table S2 Full MR results of causal links between gut microbiome and SS risk.

Group	Gut microbiota	MR method	No.SNP	OR (95% CI)	P-value
Phylum	Actinomyces	MR Egger	14	0.42 (0.11-1.65)	0.24
		Weighted median	14	0.93 (0.60-1.45)	0.74
		Inverse variance weighted	14	1.00 (0.72-1.39)	1.00
		Simple mode	14	0.69 (0.32-1.50)	0.37
		Weighted mode	14	0.80 (0.40-1.59)	0.53

Phylum	Bacteroidetes	MR Egger	10	0.59 (0.23-1.54)	0.31
		Weighted median	10	0.87 (0.51-1.49)	0.61
		Inverse variance weighted	10	0.75 (0.50-1.14)	0.18
		Simple mode	10	0.95 (0.45-2.01)	0.90
		Weighted mode	10	0.93 (0.45-1.95)	0.86
Phylum	Cyanobacteria	MR Egger	8	1.16 (0.43-3.14)	0.77
		Weighted median	8	0.96 (0.67-1.36)	0.81
		Inverse variance weighted	8	0.98 (0.75-1.30)	0.91
		Simple mode	8	0.93 (0.54-1.62)	0.82
		Weighted mode	8	0.93 (0.53-1.64)	0.82
Phylum	Euryarchaeota	MR Egger	11	0.89 (0.41-1.94)	0.77
		Weighted median	11	1.13 (0.89-1.43)	0.30
		Inverse variance weighted	11	1.05 (0.88-1.25)	0.58
		Simple mode	11	1.21 (0.83-1.78)	0.34
		Weighted mode	11	1.18 (0.82-1.70)	0.40
Phylum	Firmicutes	MR Egger	14	2.75 (1.21-6.23)	3.2e-02

		Weighted median	14	1.02 (0.66-1.58)	0.92
		Inverse variance weighted	14	1.20 (0.88-1.64)	0.25
		Simple mode	14	0.95 (0.45-1.99)	0.89
		Weighted mode	14	0.98 (0.49-1.97)	0.96
Phylum	Lentisphaerae	MR Egger	9	0.49 (0.21-1.11)	0.13
		Weighted median	9	0.79 (0.59-1.06)	0.12
		Inverse variance weighted	9	0.79 (0.63-0.99)	3.7e-02
		Simple mode	9	0.78 (0.51-1.19)	0.29
		Weighted mode	9	0.79 (0.52-1.20)	0.30
Phylum	Proteobacteria	MR Egger	12	0.36 (0.13-1.03)	0.09
		Weighted median	12	0.75 (0.46-1.21)	0.24
		Inverse variance weighted	12	0.80 (0.53-1.19)	0.27
		Simple mode	12	0.76 (0.34-1.70)	0.52
		Weighted mode	12	0.67 (0.31-1.43)	0.32
Phylum	Tenericutes	MR Egger	12	1.67 (0.63-4.41)	0.33
		Weighted median	12	0.94 (0.63-1.41)	0.78

		Inverse variance weighted	12	1.00 (0.75-1.33)	1.00
		Simple mode	12	0.94 (0.51-1.73)	0.84
		Weighted mode	12	0.94 (0.50-1.77)	0.85
Phylum	Verrucomicrobia	MR Egger	12	1.33 (0.63-2.84)	0.47
		Weighted median	12	1.12 (0.76-1.65)	0.55
		Inverse variance weighted	12	1.11 (0.84-1.49)	0.46
		Simple mode	12	1.06 (0.57-1.98)	0.85
		Weighted mode	12	1.11 (0.65-1.88)	0.72
Class	Actinobacteria	MR Egger	14	0.73 (0.33- 1.65)	0.47
		Weighted median	14	0.94 (0.64- 1.40)	0.78
		Inverse variance weighted	14	0.79 (0.60- 1.06)	0.11
		Simple mode	14	0.95 (0.52- 1.74)	0.87
		Weighted mode	14	0.94 (0.58- 1.54)	0.81
Class	Alphaproteobacteria	MR Egger	7	0.49 (0.14- 1.72)	0.32
		Weighted median	7	0.83 (0.56- 1.23)	0.35

		Inverse variance weighted	7	0.79 (0.57- 1.11)	0.18
		Simple mode	7	0.83 (0.45- 1.53)	0.57
		Weighted mode	7	0.82 (0.45- 1.50)	0.55
Class	Bacilli	MR Egger	18	0.71 (0.32- 1.55)	0.40
		Weighted median	18	0.95 (0.63- 1.43)	0.81
		Inverse variance weighted	18	1.04 (0.78- 1.38)	0.78
		Simple mode	18	0.94 (0.47- 1.90)	0.87
		Weighted mode	18	0.97 (0.51- 1.82)	0.91
Class	Bacteroidia	MR Egger	13	0.74 (0.31- 1.73)	0.50
		Weighted median	13	0.86 (0.53- 1.40)	0.55
		Inverse variance weighted	13	0.78 (0.55- 1.11)	0.17
		Simple mode	13	0.90 (0.39- 2.09)	0.81
		Weighted mode	13	0.90 (0.41- 1.98)	0.80
Class	Betaproteobacteria	MR Egger	10	2.62 (0.50-13.76)	0.29
		Weighted median	10	0.87 (0.49- 1.55)	0.65
		Inverse variance weighted	10	1.04 (0.61- 1.78)	0.87

		Simple mode	10	0.89 (0.31- 2.52)	0.83
		Weighted mode	10	1.15 (0.44- 2.97)	0.78
Class	Clostridia	MR Egger	10	1.48 (0.13-16.36)	0.76
		Weighted median	10	0.97 (0.55- 1.70)	0.92
		Inverse variance weighted	10	0.94 (0.55- 1.61)	0.83
		Simple mode	10	0.84 (0.39- 1.83)	0.68
		Weighted mode	10	0.89 (0.46- 1.72)	0.74
Class	Coriobacteriia	MR Egger	13	1.60 (0.37- 6.98)	0.54
		Weighted median	13	0.85 (0.53- 1.35)	0.49
		Inverse variance weighted	13	0.79 (0.56- 1.12)	0.18
		Simple mode	13	0.91 (0.44- 1.88)	0.80
		Weighted mode	13	0.91 (0.46- 1.80)	0.79
Class	Deltaproteobacteria	MR Egger	11	0.97 (0.15- 6.40)	0.97
		Weighted median	11	0.81 (0.50- 1.33)	0.40
		Inverse variance weighted	11	0.67 (0.47- 0.96)	3.0e-02
		Simple mode	11	0.92 (0.41- 2.09)	0.85

		Weighted mode	11	0.93 (0.42- 2.07)	0.86
Class	Erysipelotrichia	MR Egger	13	0.99 (0.11- 8.97)	0.99
		Weighted median	13	0.78 (0.46- 1.33)	0.36
		Inverse variance weighted	13	0.87 (0.54- 1.42)	0.58
		Simple mode	13	0.72 (0.30- 1.73)	0.48
		Weighted mode	13	0.75 (0.33- 1.68)	0.50
Class	Gammaproteobacteria	MR Egger	7	0.50 (0.11- 2.24)	0.41
		Weighted median	7	0.86 (0.48- 1.56)	0.63
		Inverse variance weighted	7	0.86 (0.54- 1.37)	0.53
		Simple mode	7	0.89 (0.39- 2.02)	0.79
		Weighted mode	7	0.85 (0.39- 1.86)	0.70
Class	Lentisphaeria	MR Egger	8	0.50 (0.21- 1.20)	0.17
		Weighted median	8	0.80 (0.58- 1.10)	0.17
		Inverse variance weighted	8	0.80 (0.63- 1.03)	0.08
		Simple mode	8	0.82 (0.51- 1.32)	0.45
		Weighted mode	8	0.82 (0.53- 1.28)	0.42

Class	Melainabacteria	MR Egger	10	1.88 (0.93- 3.81)	0.12
		Weighted median	10	1.19 (0.87- 1.64)	0.28
		Inverse variance weighted	10	1.00 (0.78- 1.27)	0.98
		Simple mode	10	1.26 (0.71- 2.23)	0.45
		Weighted mode	10	1.29 (0.82- 2.02)	0.30
Class	Methanobacteria	MR Egger	9	0.81 (0.31- 2.10)	0.68
		Weighted median	9	1.12 (0.84- 1.48)	0.44
		Inverse variance weighted	9	1.13 (0.89- 1.42)	0.32
		Simple mode	9	1.15 (0.74- 1.79)	0.54
		Weighted mode	9	1.13 (0.77- 1.66)	0.56
Class	Mollicutes	MR Egger	12	1.67 (0.63- 4.41)	0.33
		Weighted median	12	0.94 (0.64- 1.40)	0.78
		Inverse variance weighted	12	1.00 (0.75- 1.33)	1.00
		Simple mode	12	0.94 (0.51- 1.74)	0.84
		Weighted mode	12	0.94 (0.51- 1.73)	0.84
Class	Negativicutes	MR Egger	12	1.16 (0.34- 4.05)	0.82

		Weighted median	12	1.28 (0.79- 2.08)	0.32
		Inverse variance weighted	12	1.26 (0.88- 1.81)	0.21
		Simple mode	12	1.64 (0.71- 3.77)	0.27
		Weighted mode	12	1.61 (0.73- 3.56)	0.27
Class	Verrucomicrobiae	MR Egger	11	1.80 (0.65- 5.02)	0.29
		Weighted median	11	1.19 (0.80- 1.77)	0.39
		Inverse variance weighted	11	1.20 (0.89- 1.62)	0.24
		Simple mode	11	1.12 (0.59- 2.10)	0.74
		Weighted mode	11	1.15 (0.59- 2.21)	0.69
Order	Actinomycetales	MR Egger	4	1.01 (0.44- 2.31)	0.99
		Weighted median	4	0.97 (0.63- 1.51)	0.91
		Inverse variance weighted	4	0.95 (0.65- 1.39)	0.79
		Simple mode	4	0.98 (0.57- 1.69)	0.95
		Weighted mode	4	0.98 (0.60- 1.58)	0.93
Order	Bacillales	MR Egger	8	0.82 (0.38- 1.76)	0.63
		Weighted median	8	1.12 (0.87- 1.43)	0.39

		Inverse variance weighted	8	1.11 (0.92- 1.34)	0.27
		Simple mode	8	1.03 (0.65- 1.61)	0.91
		Weighted mode	8	1.09 (0.72- 1.66)	0.70
Order	Bacteroidales	MR Egger	13	0.74 (0.31- 1.73)	0.50
		Weighted median	13	0.86 (0.54- 1.37)	0.53
		Inverse variance weighted	13	0.78 (0.55- 1.11)	0.17
		Simple mode	13	0.90 (0.40- 2.00)	0.80
		Weighted mode	13	0.90 (0.44- 1.83)	0.77
Order	Bifidobacteriales	MR Egger	11	0.54 (0.20- 1.46)	0.25
		Weighted median	11	0.93 (0.62- 1.41)	0.75
		Inverse variance weighted	11	1.04 (0.76- 1.41)	0.82
		Simple mode	11	0.79 (0.44- 1.39)	0.43
		Weighted mode	11	0.86 (0.50- 1.46)	0.59
Order	Burkholderiales	MR Egger	10	2.91 (0.71-11.96)	0.18
		Weighted median	10	0.88 (0.50- 1.53)	0.64
		Inverse variance weighted	10	0.93 (0.57- 1.52)	0.78

		Simple mode	10	0.80 (0.30- 2.17)	0.68
		Weighted mode	10	0.90 (0.36- 2.24)	0.83
Order	Clostridiales	MR Egger	11	1.28 (0.16-10.03)	0.82
		Weighted median	11	1.00 (0.58- 1.74)	0.99
		Inverse variance weighted	11	1.25 (0.78- 2.00)	0.35
		Simple mode	11	0.83 (0.35- 1.94)	0.67
		Weighted mode	11	0.87 (0.43- 1.78)	0.71
Order	Coriobacteriales	MR Egger	13	1.60 (0.37- 6.98)	0.54
		Weighted median	13	0.85 (0.53- 1.37)	0.50
		Inverse variance weighted	13	0.79 (0.56- 1.12)	0.18
		Simple mode	13	0.91 (0.43- 1.91)	0.81
		Weighted mode	13	0.91 (0.45- 1.82)	0.80
Order	Desulfovibrionales	MR Egger	10	0.72 (0.09- 5.71)	0.76
		Weighted median	10	0.89 (0.52- 1.51)	0.66
		Inverse variance weighted	10	0.71 (0.48- 1.06)	0.10
		Simple mode	10	1.08 (0.44- 2.68)	0.86

		Weighted mode	10	1.08 (0.49- 2.36)	0.86
Order	Enterobacteriales	MR Egger	7	0.33 (0.02- 4.41)	0.44
		Weighted median	7	0.72 (0.40- 1.30)	0.27
		Inverse variance weighted	7	0.80 (0.52- 1.24)	0.32
		Simple mode	7	0.63 (0.29- 1.37)	0.29
		Weighted mode	7	0.63 (0.28- 1.43)	0.31
Order	Erysipelotrichales	MR Egger	13	0.99 (0.11- 8.97)	0.99
		Weighted median	13	0.78 (0.45- 1.34)	0.37
		Inverse variance weighted	13	0.87 (0.54- 1.42)	0.58
		Simple mode	13	0.72 (0.31- 1.67)	0.46
		Weighted mode	13	0.75 (0.34- 1.65)	0.49
Order	Gastranaerophilales	MR Egger	9	1.42 (0.69- 2.95)	0.37
		Weighted median	9	1.27 (0.92- 1.75)	0.14
		Inverse variance weighted	9	1.14 (0.89- 1.46)	0.29
		Simple mode	9	1.31 (0.82- 2.12)	0.29
		Weighted mode	9	1.32 (0.87- 2.02)	0.23

Order	Lactobacillales	MR Egger	15	0.52 (0.19- 1.44)	0.23
		Weighted median	15	1.06 (0.67- 1.67)	0.81
		Inverse variance weighted	15	1.02 (0.68- 1.52)	0.93
		Simple mode	15	1.34 (0.59- 3.04)	0.50
		Weighted mode	15	1.09 (0.52- 2.28)	0.82
Order	Methanobacteriales	MR Egger	9	0.81 (0.31- 2.10)	0.68
		Weighted median	9	1.12 (0.84- 1.48)	0.44
		Inverse variance weighted	9	1.13 (0.89- 1.42)	0.32
		Simple mode	9	1.15 (0.77- 1.73)	0.52
		Weighted mode	9	1.13 (0.78- 1.64)	0.54
Order	Mollicutes RF9	MR Egger	12	1.77 (0.64- 4.89)	0.30
		Weighted median	12	1.03 (0.70- 1.53)	0.87
		Inverse variance weighted	12	1.06 (0.75- 1.48)	0.75
		Simple mode	12	1.03 (0.51- 2.12)	0.93
		Weighted mode	12	1.03 (0.52- 2.04)	0.94
Order	NB1n	MR Egger	12	0.66 (0.30- 1.44)	0.32

		Weighted median	12	0.88 (0.68- 1.13)	0.30
		Inverse variance weighted	12	0.94 (0.78- 1.14)	0.55
		Simple mode	12	0.85 (0.58- 1.25)	0.43
		Weighted mode	12	0.84 (0.56- 1.24)	0.40
Order	Pasteurellales	MR Egger	13	1.38 (0.86- 2.21)	0.21
		Weighted median	13	1.23 (0.91- 1.66)	0.19
		Inverse variance weighted	13	1.12 (0.90- 1.40)	0.31
		Simple mode	13	1.22 (0.73- 2.06)	0.47
		Weighted mode	13	1.23 (0.79- 1.91)	0.38
Order	Rhodospirillales	MR Egger	14	0.67 (0.23- 1.95)	0.48
		Weighted median	14	0.85 (0.62- 1.16)	0.30
		Inverse variance weighted	14	0.90 (0.70- 1.17)	0.44
		Simple mode	14	0.79 (0.45- 1.40)	0.44
		Weighted mode	14	0.83 (0.50- 1.35)	0.46
Order	Selenomonadales	MR Egger	12	1.16 (0.34- 4.05)	0.82
		Weighted median	12	1.28 (0.78- 2.09)	0.32

		Inverse variance weighted	12	1.26 (0.88- 1.81)	0.21
		Simple mode	12	1.64 (0.71- 3.78)	0.27
		Weighted mode	12	1.61 (0.70- 3.68)	0.29
Order	Verrucomicrobiales	MR Egger	11	1.80 (0.65- 5.02)	0.29
		Weighted median	11	1.19 (0.79- 1.79)	0.41
		Inverse variance weighted	11	1.20 (0.89- 1.62)	0.24
		Simple mode	11	1.12 (0.56- 2.21)	0.76
		Weighted mode	11	1.15 (0.60- 2.17)	0.68
Order	Victivallales	MR Egger	8	0.50 (0.21- 1.20)	0.17
		Weighted median	8	0.80 (0.59- 1.09)	0.16
		Inverse variance weighted	8	0.80 (0.63- 1.03)	0.08
		Simple mode	8	0.82 (0.53- 1.28)	0.42
		Weighted mode	8	0.82 (0.55- 1.24)	0.39
Family	Acidaminococcaceae	MR Egger	7	0.65 (0.23- 1.89)	0.47
		Weighted median	7	1.10 (0.70- 1.73)	0.68
		Inverse variance weighted	7	1.03 (0.73- 1.46)	0.87

		Simple mode	7	1.16 (0.60- 2.25)	0.68
		Weighted mode	7	1.16 (0.57- 2.37)	0.70
Family	Actinomycetaceae	MR Egger	4	1.01 (0.44- 2.30)	0.99
		Weighted median	4	0.97 (0.63- 1.51)	0.91
		Inverse variance weighted	4	0.95 (0.65- 1.39)	0.79
		Simple mode	4	0.98 (0.54- 1.78)	0.95
		Weighted mode	4	0.98 (0.59- 1.61)	0.93
Family	Alcaligenaceae	MR Egger	11	3.19 (0.56-18.07)	0.22
		Weighted median	11	0.87 (0.53- 1.43)	0.58
		Inverse variance weighted	11	0.84 (0.58- 1.23)	0.38
		Simple mode	11	0.90 (0.42- 1.95)	0.79
		Weighted mode	11	0.91 (0.43- 1.94)	0.81
Family	Bacteroidaceae	MR Egger	7	8.86 (0.93-84.09)	0.12
		Weighted median	7	0.94 (0.50- 1.77)	0.86
		Inverse variance weighted	7	0.75 (0.44- 1.28)	0.28
		Simple mode	7	1.09 (0.40- 2.99)	0.87

		Weighted mode	7	1.02 (0.45- 2.32)	0.96
Family	Bacteroidales (S24.7 group)	MR Egger	8	1.28 (0.26- 6.27)	0.77
		Weighted median	8	1.17 (0.78- 1.77)	0.45
		Inverse variance weighted	8	1.17 (0.81- 1.69)	0.40
		Simple mode	8	1.12 (0.53- 2.37)	0.77
		Weighted mode	8	1.15 (0.61- 2.17)	0.69
Family	Bifidobacteriaceae	MR Egger	11	0.54 (0.20- 1.46)	0.25
		Weighted median	11	0.93 (0.61- 1.42)	0.75
		Inverse variance weighted	11	1.04 (0.76- 1.41)	0.82
		Simple mode	11	0.79 (0.42- 1.46)	0.46
		Weighted mode	11	0.86 (0.53- 1.38)	0.54
Family	Christensenellaceae	MR Egger	11	0.83 (0.46- 1.52)	0.56
		Weighted median	11	1.10 (0.74- 1.64)	0.64
		Inverse variance weighted	11	1.09 (0.80- 1.49)	0.59
		Simple mode	11	0.77 (0.39- 1.50)	0.46
		Weighted mode	11	1.07 (0.68- 1.68)	0.78

Family	Clostridiaceae 1	MR Egger	10	1.16 (0.43- 3.16)	0.78
		Weighted median	10	1.15 (0.73- 1.81)	0.54
		Inverse variance weighted	10	0.97 (0.69- 1.37)	0.86
		Simple mode	10	1.27 (0.62- 2.60)	0.53
		Weighted mode	10	1.26 (0.67- 2.38)	0.49
Family	Clostridialesvadin (BB60 group)	MR Egger	15	1.39 (0.72- 2.69)	0.35
		Weighted median	15	1.14 (0.81- 1.59)	0.45
		Inverse variance weighted	15	1.21 (0.95- 1.53)	0.13
		Simple mode	15	1.22 (0.73- 2.05)	0.45
		Weighted mode	15	1.17 (0.73- 1.87)	0.53
Family	Coriobacteriaceae	MR Egger	13	1.60 (0.37- 6.98)	0.54
		Weighted median	13	0.85 (0.54- 1.32)	0.47
		Inverse variance weighted	13	0.79 (0.56- 1.12)	0.18
		Simple mode	13	0.91 (0.42- 1.97)	0.82
		Weighted mode	13	0.91 (0.44- 1.88)	0.80
Family	Defluviitaleaceae	MR Egger	11	0.92 (0.37- 2.31)	0.86

		Weighted median	11	0.92 (0.64- 1.34)	0.68
		Inverse variance weighted	11	0.97 (0.74- 1.27)	0.83
		Simple mode	11	0.67 (0.33- 1.34)	0.28
		Weighted mode	11	0.70 (0.38- 1.29)	0.27
Family	Desulfovibrionaceae	MR Egger	8	1.01 (0.10-10.61)	0.99
		Weighted median	8	0.82 (0.45- 1.47)	0.50
		Inverse variance weighted	8	0.65 (0.41- 1.02)	0.06
		Simple mode	8	0.98 (0.37- 2.60)	0.96
		Weighted mode	8	1.01 (0.45- 2.26)	0.98
Family	Enterobacteriaceae	MR Egger	7	0.33 (0.02- 4.41)	0.44
		Weighted median	7	0.72 (0.41- 1.26)	0.25
		Inverse variance weighted	7	0.80 (0.52- 1.24)	0.32
		Simple mode	7	0.63 (0.27- 1.47)	0.33
		Weighted mode	7	0.63 (0.28- 1.40)	0.30
Family	Erysipelotrichaceae	MR Egger	13	0.99 (0.11- 8.97)	0.99
		Weighted median	13	0.78 (0.46- 1.32)	0.35

		Inverse variance weighted	13	0.87 (0.54- 1.42)	0.58
		Simple mode	13	0.72 (0.31- 1.70)	0.47
		Weighted mode	13	0.75 (0.35- 1.60)	0.47
Family	Family XI	MR Egger	8	3.67 (1.13-11.95)	0.07
		Weighted median	8	0.98 (0.76- 1.28)	0.91
		Inverse variance weighted	8	0.98 (0.78- 1.23)	0.84
		Simple mode	8	1.01 (0.66- 1.53)	0.97
		Weighted mode	8	1.01 (0.68- 1.50)	0.96
Family	Family XIII	MR Egger	7	0.11 (0.02- 0.73)	0.07
		Weighted median	7	0.96 (0.51- 1.81)	0.89
		Inverse variance weighted	7	0.85 (0.47- 1.51)	0.57
		Simple mode	7	0.95 (0.41- 2.20)	0.91
		Weighted mode	7	0.95 (0.42- 2.13)	0.91
Family	Lachnospiraceae	MR Egger	16	0.33 (0.08- 1.44)	0.16
		Weighted median	16	1.22 (0.75- 1.99)	0.43
		Inverse variance weighted	16	1.09 (0.70- 1.70)	0.70

		Simple mode	16	1.01 (0.41- 2.50)	0.98
		Weighted mode	16	1.24 (0.54- 2.86)	0.62
Family	Lactobacillaceae	MR Egger	8	1.18 (0.63- 2.20)	0.63
		Weighted median	8	0.79 (0.56- 1.11)	0.18
		Inverse variance weighted	8	0.88 (0.68- 1.13)	0.30
		Simple mode	8	0.74 (0.46- 1.20)	0.27
		Weighted mode	8	0.75 (0.50- 1.13)	0.22
Family	Methanobacteriaceae	MR Egger	9	0.81 (0.31- 2.10)	0.68
		Weighted median	9	1.12 (0.84- 1.48)	0.44
		Inverse variance weighted	9	1.13 (0.89- 1.42)	0.32
		Simple mode	9	1.15 (0.76- 1.76)	0.53
		Weighted mode	9	1.13 (0.77- 1.65)	0.55
Family	Oxalobacteraceae	MR Egger	14	0.84 (0.43- 1.65)	0.62
		Weighted median	14	0.89 (0.71- 1.12)	0.32
		Inverse variance weighted	14	0.91 (0.76- 1.08)	0.29
		Simple mode	14	0.83 (0.56- 1.22)	0.35

		Weighted mode	14	0.81 (0.56- 1.17)	0.29
Family	Pasteurellaceae	MR Egger	7	8.86 (0.93-84.09)	0.12
		Weighted median	7	0.94 (0.51- 1.76)	0.86
		Inverse variance weighted	7	0.75 (0.44- 1.28)	0.28
		Simple mode	7	1.09 (0.40- 3.01)	0.87
		Weighted mode	7	1.02 (0.46- 2.29)	0.96
Family	Peptococcaceae	MR Egger	9	1.32 (0.27- 6.44)	0.74
		Weighted median	9	1.14 (0.73- 1.78)	0.57
		Inverse variance weighted	9	1.12 (0.66- 1.91)	0.68
		Simple mode	9	1.09 (0.48- 2.52)	0.84
		Weighted mode	9	1.20 (0.64- 2.25)	0.59
Family	Peptostreptococcaceae	MR Egger	11	0.54 (0.20- 1.46)	0.25
		Weighted median	11	0.93 (0.63- 1.39)	0.74
		Inverse variance weighted	11	1.04 (0.76- 1.41)	0.82
		Simple mode	11	0.79 (0.42- 1.46)	0.46
		Weighted mode	11	0.86 (0.51- 1.45)	0.58

Family	Porphyromonadaceae	MR Egger	9	0.15 (0.02- 1.12)	0.11
		Weighted median	9	0.80 (0.45- 1.43)	0.45
		Inverse variance weighted	9	0.60 (0.38- 0.94)	2.6e-02
		Simple mode	9	0.80 (0.31- 2.05)	0.65
		Weighted mode	9	0.85 (0.34- 2.11)	0.73
Family	Prevotellaceae	MR Egger	15	0.92 (0.30- 2.86)	0.89
		Weighted median	15	0.94 (0.62- 1.43)	0.78
		Inverse variance weighted	15	1.09 (0.80- 1.49)	0.58
		Simple mode	15	0.77 (0.34- 1.77)	0.55
		Weighted mode	15	0.74 (0.36- 1.53)	0.43
Family	Rhodospirillaceae	MR Egger	15	0.70 (0.23- 2.12)	0.54
		Weighted median	15	0.91 (0.67- 1.24)	0.55
		Inverse variance weighted	15	0.94 (0.73- 1.22)	0.65
		Simple mode	15	1.01 (0.56- 1.83)	0.97
		Weighted mode	15	0.91 (0.56- 1.48)	0.72
Family	Rikenellaceae	MR Egger	16	1.25 (0.50- 3.15)	0.65

		Weighted median	16	0.76 (0.51- 1.14)	0.19
		Inverse variance weighted	16	0.84 (0.62- 1.14)	0.26
		Simple mode	16	0.67 (0.32- 1.41)	0.31
		Weighted mode	16	0.69 (0.34- 1.39)	0.32
Family	Ruminococcaceae	MR Egger	9	1.44 (0.64- 3.27)	0.41
		Weighted median	9	0.93 (0.55- 1.57)	0.78
		Inverse variance weighted	9	0.97 (0.68- 1.40)	0.89
		Simple mode	9	0.61 (0.25- 1.50)	0.31
		Weighted mode	9	0.60 (0.23- 1.56)	0.33
Family	Streptococcaceae	MR Egger	11	0.54 (0.05- 5.97)	0.63
		Weighted median	11	0.78 (0.44- 1.36)	0.38
		Inverse variance weighted	11	0.77 (0.44- 1.34)	0.36
		Simple mode	11	0.59 (0.25- 1.38)	0.25
		Weighted mode	11	0.65 (0.33- 1.31)	0.26
Family	unknownfamily	MR Egger	9	1.42 (0.69- 2.95)	0.37
		Weighted median	9	1.27 (0.92- 1.75)	0.15

		Inverse variance weighted	9	1.14 (0.89- 1.46)	0.29
		Simple mode	9	1.31 (0.81- 2.14)	0.30
		Weighted mode	9	1.32 (0.87- 2.02)	0.23
Family	unknownfamily	MR Egger	12	1.77 (0.64- 4.89)	0.30
		Weighted median	12	1.03 (0.70- 1.53)	0.87
		Inverse variance weighted	12	1.06 (0.75- 1.48)	0.75
		Simple mode	12	1.03 (0.52- 2.07)	0.92
		Weighted mode	12	1.03 (0.52- 2.04)	0.94
Family	unknownfamily	MR Egger	12	0.66 (0.30- 1.44)	0.32
		Weighted median	12	0.88 (0.68- 1.12)	0.29
		Inverse variance weighted	12	0.94 (0.78- 1.14)	0.55
		Simple mode	12	0.85 (0.58- 1.25)	0.43
		Weighted mode	12	0.84 (0.56- 1.25)	0.41
Family	Veillonellaceae	MR Egger	19	1.12 (0.68- 1.83)	0.66
		Weighted median	19	1.13 (0.78- 1.62)	0.53
		Inverse variance weighted	19	0.96 (0.75- 1.23)	0.75

		Simple mode	19	1.30 (0.66- 2.55)	0.46
		Weighted mode	19	1.28 (0.76- 2.15)	0.36
Family	Verrucomicrobiaceae	MR Egger	11	1.80 (0.65- 5.02)	0.29
		Weighted median	11	1.19 (0.80- 1.77)	0.40
		Inverse variance weighted	11	1.20 (0.89- 1.62)	0.24
		Simple mode	11	1.12 (0.56- 2.24)	0.77
		Weighted mode	11	1.15 (0.60- 2.20)	0.69
Family	Victivallaceae	MR Egger	12	0.77 (0.35- 1.69)	0.53
		Weighted median	12	1.06 (0.85- 1.33)	0.58
		Inverse variance weighted	12	1.10 (0.94- 1.30)	0.24
		Simple mode	12	1.23 (0.89- 1.72)	0.24
		Weighted mode	12	1.21 (0.85- 1.71)	0.31
Genus	Clostridium (innocuum group)	MR Egger	7	0.57 (0.18- 1.75)	0.37
		Weighted median	7	1.15 (0.86- 1.54)	0.34
		Inverse variance weighted	7	1.14 (0.90- 1.45)	0.27
		Simple mode	7	1.06 (0.71- 1.57)	0.80

		Weighted mode	7	1.10 (0.77- 1.58)	0.61
Genus	Eubacterium (brachy group)	MR Egger	10	1.25 (0.39- 4.02)	0.72
		Weighted median	10	1.25 (0.92- 1.69)	0.15
		Inverse variance weighted	10	1.26 (0.96- 1.66)	0.10
		Simple mode	10	1.35 (0.77- 2.38)	0.32
		Weighted mode	10	1.30 (0.78- 2.15)	0.34
Genus	Eubacterium (coprostanoligenes group)	MR Egger	13	0.18 (0.04- 0.83)	0.05
		Weighted median	13	0.55 (0.32- 0.93)	2.6e-02
		Inverse variance weighted	13	0.61 (0.40- 0.93)	2.1e-02
		Simple mode	13	0.53 (0.22- 1.28)	0.18
		Weighted mode	13	0.52 (0.22- 1.21)	0.16
Genus	Eubacterium (eligens group)	MR Egger	6	1.48 (0.26- 8.33)	0.68
		Weighted median	6	1.25 (0.72- 2.20)	0.43
		Inverse variance weighted	6	1.18 (0.74- 1.87)	0.49
		Simple mode	6	1.33 (0.65- 2.73)	0.47
		Weighted mode	6	1.30 (0.63- 2.67)	0.51

Genus	Eubacterium (fissicatena group)	MR Egger	9	0.77 (0.15- 4.10)	0.77
		Weighted median	9	1.13 (0.82- 1.57)	0.45
		Inverse variance weighted	9	1.04 (0.77- 1.41)	0.79
		Simple mode	9	1.27 (0.69- 2.37)	0.47
		Weighted mode	9	1.30 (0.71- 2.35)	0.42
Genus	Eubacterium (hallii group)	MR Egger	15	1.21 (0.67- 2.18)	0.53
		Weighted median	15	0.97 (0.67- 1.42)	0.89
		Inverse variance weighted	15	0.92 (0.70- 1.22)	0.57
		Simple mode	15	0.83 (0.43- 1.57)	0.57
		Weighted mode	15	1.02 (0.58- 1.79)	0.94
Genus	Eubacterium (nodatum group)	MR Egger	11	1.60 (0.75- 3.40)	0.25
		Weighted median	11	0.99 (0.79- 1.25)	0.96
		Inverse variance weighted	11	0.98 (0.83- 1.16)	0.84
		Simple mode	11	1.03 (0.75- 1.41)	0.85
		Weighted mode	11	1.03 (0.77- 1.38)	0.84
Genus	Eubacterium (oxidoreducens group)	MR Egger	5	0.80 (0.25- 2.53)	0.73

		Weighted median	5	1.12 (0.76- 1.65)	0.56
		Inverse variance weighted	5	1.11 (0.81- 1.51)	0.53
		Simple mode	5	1.10 (0.66- 1.85)	0.73
		Weighted mode	5	1.10 (0.66- 1.83)	0.74
Genus	Eubacterium (rectale group)	MR Egger	8	0.55 (0.12- 2.63)	0.48
		Weighted median	8	0.74 (0.42- 1.30)	0.29
		Inverse variance weighted	8	0.83 (0.54- 1.27)	0.39
		Simple mode	8	0.59 (0.24- 1.45)	0.29
		Weighted mode	8	0.58 (0.25- 1.37)	0.26
Genus	Eubacterium (ruminantium group)	MR Egger	18	0.95 (0.46- 1.93)	0.88
		Weighted median	18	1.03 (0.78- 1.36)	0.81
		Inverse variance weighted	18	0.95 (0.77- 1.17)	0.62
		Simple mode	18	1.07 (0.66- 1.74)	0.79
		Weighted mode	18	1.07 (0.69- 1.66)	0.77
Genus	Eubacterium (ventriosum group)	MR Egger	15	1.88 (0.49- 7.13)	0.37
		Weighted median	15	0.84 (0.57- 1.25)	0.40

		Inverse variance weighted	15	0.82 (0.61- 1.10)	0.19
		Simple mode	15	0.72 (0.35- 1.45)	0.37
		Weighted mode	15	1.15 (0.58- 2.29)	0.69
Genus	Eubacterium (xylanophilum group)	MR Egger	9	0.82 (0.30- 2.20)	0.71
		Weighted median	9	1.07 (0.69- 1.66)	0.76
		Inverse variance weighted	9	1.03 (0.74- 1.44)	0.84
		Simple mode	9	1.14 (0.57- 2.27)	0.73
		Weighted mode	9	1.04 (0.56- 1.91)	0.91
Genus	Ruminococcus (gauvreauii group)	MR Egger	11	0.73 (0.12- 4.42)	0.74
		Weighted median	11	1.17 (0.75- 1.83)	0.48
		Inverse variance weighted	11	1.23 (0.81- 1.87)	0.34
		Simple mode	11	1.10 (0.57- 2.14)	0.77
		Weighted mode	11	1.11 (0.58- 2.16)	0.75
Genus	Ruminococcus (gnavus group)	MR Egger	11	1.67 (0.54- 5.15)	0.40
		Weighted median	11	1.06 (0.78- 1.45)	0.71
		Inverse variance weighted	11	0.91 (0.72- 1.16)	0.46

		Simple mode	11	1.17 (0.72- 1.90)	0.54
		Weighted mode	11	1.14 (0.74- 1.77)	0.57
Genus	Ruminococcus (torques group)	MR Egger	7	0.87 (0.17- 4.42)	0.87
		Weighted median	7	1.24 (0.62- 2.47)	0.55
		Inverse variance weighted	7	1.02 (0.62- 1.69)	0.93
		Simple mode	7	1.25 (0.45- 3.48)	0.68
		Weighted mode	7	1.27 (0.49- 3.34)	0.64
Genus	Actinomyces	MR Egger	7	0.99 (0.48- 2.02)	0.97
		Weighted median	7	0.96 (0.66- 1.40)	0.83
		Inverse variance weighted	7	0.92 (0.69- 1.24)	0.60
		Simple mode	7	0.97 (0.61- 1.57)	0.92
		Weighted mode	7	0.96 (0.62- 1.49)	0.87
Genus	Adlercreutzia	MR Egger	8	0.24 (0.06- 0.99)	0.10
		Weighted median	8	0.81 (0.52- 1.26)	0.35
		Inverse variance weighted	8	0.98 (0.68- 1.42)	0.93
		Simple mode	8	0.66 (0.28- 1.54)	0.37

		Weighted mode	8	0.65 (0.30- 1.41)	0.32
Genus	Akkermansia	MR Egger	11	1.80 (0.65- 5.01)	0.29
		Weighted median	11	1.19 (0.81- 1.74)	0.38
		Inverse variance weighted	11	1.20 (0.89- 1.62)	0.24
		Simple mode	11	1.11 (0.58- 2.15)	0.75
		Weighted mode	11	1.14 (0.59- 2.24)	0.70
Genus	Alistipes	MR Egger	12	0.36 (0.02- 7.68)	0.53
		Weighted median	12	0.77 (0.44- 1.33)	0.34
		Inverse variance weighted	12	0.89 (0.48- 1.65)	0.71
		Simple mode	12	0.76 (0.34- 1.74)	0.53
		Weighted mode	12	0.75 (0.33- 1.67)	0.49
Genus	Allisonella	MR Egger	8	0.80 (0.23- 2.81)	0.74
		Weighted median	8	0.94 (0.76- 1.17)	0.59
		Inverse variance weighted	8	0.94 (0.78- 1.13)	0.53
		Simple mode	8	0.93 (0.68- 1.28)	0.67
		Weighted mode	8	0.94 (0.68- 1.30)	0.73

Genus	Alloprevotella	MR Egger	6	1.48 (0.26- 8.33)	0.68
		Weighted median	6	1.25 (0.71- 2.21)	0.43
		Inverse variance weighted	6	1.18 (0.74- 1.87)	0.49
		Simple mode	6	1.33 (0.63- 2.82)	0.49
		Weighted mode	6	1.30 (0.64- 2.61)	0.50
Genus	Anaerofilum	MR Egger	11	0.96 (0.27- 3.47)	0.95
		Weighted median	11	0.90 (0.67- 1.22)	0.51
		Inverse variance weighted	11	0.96 (0.77- 1.20)	0.71
		Simple mode	11	0.83 (0.51- 1.34)	0.46
		Weighted mode	11	0.86 (0.54- 1.35)	0.53
Genus	Anaerostipes	MR Egger	15	1.21 (0.67- 2.18)	0.53
		Weighted median	15	0.97 (0.66- 1.43)	0.89
		Inverse variance weighted	15	0.92 (0.70- 1.22)	0.57
		Simple mode	15	0.83 (0.43- 1.58)	0.57
		Weighted mode	15	1.02 (0.59- 1.77)	0.94
Genus	Anaerotruncus	MR Egger	13	0.53 (0.17- 1.61)	0.29

		Weighted median	13	0.91 (0.55- 1.50)	0.70
		Inverse variance weighted	13	1.12 (0.75- 1.66)	0.59
		Simple mode	13	0.82 (0.34- 1.99)	0.67
		Weighted mode	13	0.81 (0.38- 1.74)	0.61
Genus	Bacteroides	MR Egger	7	8.86 (0.93-84.09)	0.12
		Weighted median	7	0.94 (0.50- 1.79)	0.86
		Inverse variance weighted	7	0.75 (0.44- 1.28)	0.28
		Simple mode	7	1.09 (0.45- 2.66)	0.85
		Weighted mode	7	1.02 (0.43- 2.41)	0.96
Genus	Barnesiella	MR Egger	12	0.93 (0.24- 3.55)	0.92
		Weighted median	12	1.22 (0.78- 1.91)	0.39
		Inverse variance weighted	12	1.19 (0.86- 1.66)	0.29
		Simple mode	12	0.98 (0.46- 2.10)	0.97
		Weighted mode	12	1.05 (0.53- 2.06)	0.89
Genus	Bifidobacterium	MR Egger	12	0.81 (0.41- 1.62)	0.57
		Weighted median	12	0.98 (0.69- 1.40)	0.90

		Inverse variance weighted	12	1.06 (0.80- 1.40)	0.67
		Simple mode	12	0.91 (0.53- 1.56)	0.74
		Weighted mode	12	0.96 (0.63- 1.44)	0.83
Genus	Bilophila	MR Egger	13	0.61 (0.09- 4.14)	0.62
		Weighted median	13	0.75 (0.48- 1.18)	0.21
		Inverse variance weighted	13	0.93 (0.64- 1.35)	0.69
		Simple mode	13	0.61 (0.27- 1.38)	0.26
		Weighted mode	13	0.61 (0.29- 1.29)	0.22
Genus	Blautia	MR Egger	12	1.19 (0.46- 3.07)	0.73
		Weighted median	12	0.70 (0.42- 1.16)	0.16
		Inverse variance weighted	12	0.62 (0.43- 0.90)	1.2e-02
		Simple mode	12	0.83 (0.35- 1.95)	0.68
		Weighted mode	12	0.88 (0.39- 1.96)	0.75
Genus	Butyricicoccus	MR Egger	8	0.47 (0.22- 1.01)	0.10
		Weighted median	8	0.58 (0.35- 0.95)	3.2e-02
		Inverse variance weighted	8	0.61 (0.42- 0.90)	1.2e-02

		Simple mode	8	0.52 (0.25- 1.08)	0.12
		Weighted mode	8	0.55 (0.29- 1.04)	0.11
Genus	Butyricimonas	MR Egger	13	1.02 (0.34- 3.01)	0.98
		Weighted median	13	1.12 (0.76- 1.66)	0.56
		Inverse variance weighted	13	1.06 (0.79- 1.43)	0.68
		Simple mode	13	1.12 (0.61- 2.04)	0.73
		Weighted mode	13	1.10 (0.61- 1.99)	0.75
Genus	Butyrivibrio	MR Egger	15	0.75 (0.41- 1.38)	0.37
		Weighted median	15	0.93 (0.77- 1.14)	0.51
		Inverse variance weighted	15	0.92 (0.80- 1.06)	0.23
		Simple mode	15	1.01 (0.74- 1.39)	0.95
		Weighted mode	15	1.00 (0.73- 1.38)	0.99
Genus	CandidatusSoleaferrea	MR Egger	9	0.15 (0.01- 1.80)	0.18
		Weighted median	9	1.16 (0.81- 1.66)	0.41
		Inverse variance weighted	9	1.19 (0.92- 1.53)	0.18
		Simple mode	9	1.11 (0.62- 2.00)	0.74

		Weighted mode	9	1.11 (0.60- 2.05)	0.75
Genus	Catenibacterium	MR Egger	4	1.52 (0.05-49.83)	0.84
		Weighted median	4	0.90 (0.64- 1.27)	0.55
		Inverse variance weighted	4	0.88 (0.67- 1.16)	0.37
		Simple mode	4	0.75 (0.47- 1.20)	0.32
		Weighted mode	4	1.03 (0.62- 1.72)	0.91
Genus	Christensenellaceae (R.7 group)	MR Egger	8	0.27 (0.06- 1.32)	0.16
		Weighted median	8	0.82 (0.45- 1.49)	0.52
		Inverse variance weighted	8	0.83 (0.53- 1.30)	0.41
		Simple mode	8	0.86 (0.32- 2.31)	0.77
		Weighted mode	8	0.85 (0.31- 2.35)	0.76
Genus	Clostridiumsensustricto 1	MR Egger	7	0.80 (0.24- 2.59)	0.72
		Weighted median	7	1.31 (0.81- 2.13)	0.27
		Inverse variance weighted	7	1.11 (0.72- 1.71)	0.64
		Simple mode	7	1.43 (0.73- 2.81)	0.34
		Weighted mode	7	1.36 (0.74- 2.48)	0.36

Genus	Collinsella	MR Egger	9	1.59 (0.35- 7.30)	0.57
		Weighted median	9	0.98 (0.56- 1.72)	0.94
		Inverse variance weighted	9	0.98 (0.65- 1.47)	0.91
		Simple mode	9	0.75 (0.31- 1.79)	0.53
		Weighted mode	9	0.72 (0.29- 1.77)	0.49
Genus	Coprobacter	MR Egger	10	0.57 (0.19- 1.68)	0.34
		Weighted median	10	1.11 (0.79- 1.57)	0.56
		Inverse variance weighted	10	1.09 (0.81- 1.46)	0.58
		Simple mode	10	1.13 (0.60- 2.14)	0.71
		Weighted mode	10	1.11 (0.59- 2.09)	0.75
Genus	Coprococcus 1	MR Egger	11	1.01 (0.44- 2.32)	0.97
		Weighted median	11	1.16 (0.72- 1.86)	0.55
		Inverse variance weighted	11	1.00 (0.72- 1.41)	0.98
		Simple mode	11	1.18 (0.59- 2.35)	0.65
		Weighted mode	11	1.21 (0.65- 2.25)	0.55
Genus	Coprococcus 2	MR Egger	8	3.75 (0.21-68.49)	0.41

		Weighted median	8	1.05 (0.67- 1.65)	0.84
		Inverse variance weighted	8	1.12 (0.78- 1.62)	0.54
		Simple mode	8	1.04 (0.51- 2.10)	0.92
		Weighted mode	8	1.04 (0.51- 2.12)	0.92
Genus	Coprococcus 3	MR Egger	9	0.82 (0.08- 8.17)	0.87
		Weighted median	9	0.77 (0.44- 1.37)	0.38
		Inverse variance weighted	9	0.78 (0.51- 1.18)	0.23
		Simple mode	9	0.79 (0.34- 1.85)	0.60
		Weighted mode	9	0.79 (0.38- 1.64)	0.54
Genus	Defluviitaleaceae (UCG011)	MR Egger	9	0.65 (0.18- 2.31)	0.53
		Weighted median	9	0.70 (0.45- 1.08)	0.10
		Inverse variance weighted	9	0.92 (0.66- 1.29)	0.64
		Simple mode	9	0.64 (0.35- 1.19)	0.20
		Weighted mode	9	0.66 (0.36- 1.21)	0.22
Genus	Desulfovibrio	MR Egger	10	0.88 (0.36- 2.16)	0.79
		Weighted median	10	0.93 (0.61- 1.40)	0.71

		Inverse variance weighted	10	0.91 (0.68- 1.22)	0.53
		Simple mode	10	0.96 (0.53- 1.74)	0.89
		Weighted mode	10	0.95 (0.49- 1.83)	0.88
Genus	Dialister	MR Egger	11	0.21 (0.04- 1.22)	0.12
		Weighted median	11	0.71 (0.44- 1.13)	0.15
		Inverse variance weighted	11	0.93 (0.58- 1.49)	0.77
		Simple mode	11	0.55 (0.24- 1.29)	0.20
		Weighted mode	11	0.56 (0.24- 1.32)	0.21
Genus	Dorea	MR Egger	10	1.25 (0.29- 5.40)	0.77
		Weighted median	10	0.90 (0.50- 1.63)	0.73
		Inverse variance weighted	10	0.85 (0.52- 1.42)	0.54
		Simple mode	10	0.54 (0.16- 1.77)	0.34
		Weighted mode	10	1.21 (0.47- 3.09)	0.70
Genus	Eggerthella	MR Egger	10	0.32 (0.11- 0.89)	0.06
		Weighted median	10	1.16 (0.85- 1.58)	0.34
		Inverse variance weighted	10	1.00 (0.78- 1.28)	0.97

		Simple mode	10	1.20 (0.78- 1.87)	0.43
		Weighted mode	10	1.21 (0.76- 1.92)	0.44
Genus	Eisenbergiella	MR Egger	11	0.50 (0.07- 3.63)	0.51
		Weighted median	11	1.03 (0.75- 1.39)	0.87
		Inverse variance weighted	11	1.05 (0.81- 1.37)	0.69
		Simple mode	11	1.06 (0.64- 1.77)	0.82
		Weighted mode	11	1.06 (0.66- 1.72)	0.81
Genus	Enterorhabdus	MR Egger	6	1.05 (0.43- 2.60)	0.91
		Weighted median	6	1.09 (0.70- 1.70)	0.70
		Inverse variance weighted	6	1.21 (0.86- 1.70)	0.27
		Simple mode	6	1.05 (0.57- 1.95)	0.87
		Weighted mode	6	1.06 (0.62- 1.83)	0.84
Genus	Erysipelatoclostridium	MR Egger	15	1.17 (0.31- 4.41)	0.82
		Weighted median	15	0.75 (0.53- 1.08)	0.13
		Inverse variance weighted	15	0.92 (0.66- 1.27)	0.61
		Simple mode	15	0.64 (0.30- 1.37)	0.27

		Weighted mode	15	0.70 (0.36- 1.38)	0.32
Genus	Erysipelotrichaceae (UCG003)	MR Egger	16	1.06 (0.43- 2.60)	0.91
		Weighted median	16	1.00 (0.68- 1.47)	1.00
		Inverse variance weighted	16	0.98 (0.71- 1.35)	0.89
		Simple mode	16	0.89 (0.41- 1.94)	0.77
		Weighted mode	16	0.87 (0.42- 1.82)	0.72
Genus	Escherichia Shigella	MR Egger	10	0.86 (0.28- 2.64)	0.79
		Weighted median	10	0.67 (0.42- 1.07)	0.09
		Inverse variance weighted	10	0.70 (0.49- 0.99)	4.5e-02
		Simple mode	10	0.57 (0.25- 1.32)	0.22
		Weighted mode	10	0.61 (0.28- 1.33)	0.25
Genus	Faecalibacterium	MR Egger	10	1.42 (0.78- 2.58)	0.29
		Weighted median	10	1.06 (0.69- 1.65)	0.78
		Inverse variance weighted	10	0.93 (0.68- 1.26)	0.64
		Simple mode	10	0.55 (0.25- 1.17)	0.15
		Weighted mode	10	1.16 (0.70- 1.91)	0.58

Genus	Family XIIIAD (3011 group)	MR Egger	13	0.35 (0.01- 17.01)	0.61
		Weighted median	13	0.63 (0.40- 0.98)	4.2e-02
		Inverse variance weighted	13	0.53 (0.24- 1.16)	0.11
		Simple mode	13	0.58 (0.30- 1.13)	0.14
		Weighted mode	13	0.58 (0.30- 1.15)	0.15
Genus	FamilyXIIIUCG001	MR Egger	8	0.45 (0.15- 1.40)	0.22
		Weighted median	8	1.00 (0.62- 1.63)	0.99
		Inverse variance weighted	8	1.09 (0.76- 1.58)	0.63
		Simple mode	8	0.94 (0.45- 1.97)	0.87
		Weighted mode	8	0.93 (0.43- 2.00)	0.85
Genus	Flavonifractor	MR Egger	5	0.52 (0.09- 2.89)	0.51
		Weighted median	5	0.91 (0.54- 1.55)	0.74
		Inverse variance weighted	5	0.99 (0.64- 1.52)	0.95
		Simple mode	5	0.87 (0.44- 1.70)	0.70
		Weighted mode	5	0.87 (0.44- 1.72)	0.71
Genus	Fusicatenibacter	MR Egger	18	1.86 (0.61- 5.66)	0.29

		Weighted median	18	1.32 (0.89- 1.97)	0.17
		Inverse variance weighted	18	1.31 (0.97- 1.76)	0.08
		Simple mode	18	1.40 (0.65- 3.03)	0.41
		Weighted mode	18	1.42 (0.63- 3.16)	0.41
Genus	Gordonibacter	MR Egger	10	1.22 (0.49- 3.00)	0.68
		Weighted median	10	0.88 (0.69- 1.13)	0.32
		Inverse variance weighted	10	0.82 (0.66- 1.03)	0.09
		Simple mode	10	0.89 (0.61- 1.30)	0.56
		Weighted mode	10	0.89 (0.63- 1.26)	0.54
Genus	Haemophilus	MR Egger	9	0.96 (0.41- 2.24)	0.93
		Weighted median	9	1.22 (0.84- 1.78)	0.30
		Inverse variance weighted	9	1.04 (0.73- 1.49)	0.81
		Simple mode	9	1.12 (0.62- 2.05)	0.71
		Weighted mode	9	1.16 (0.73- 1.84)	0.55
Genus	Holdemanella	MR Egger	11	1.42 (0.74- 2.70)	0.32
		Weighted median	11	1.31 (0.98- 1.76)	0.07

		Inverse variance weighted	11	1.24 (0.99- 1.55)	0.06
		Simple mode	11	1.35 (0.87- 2.09)	0.21
		Weighted mode	11	1.38 (0.91- 2.08)	0.16
Genus	Holdemania	MR Egger	14	1.09 (0.50- 2.38)	0.83
		Weighted median	14	0.97 (0.69- 1.37)	0.85
		Inverse variance weighted	14	0.90 (0.69- 1.16)	0.41
		Simple mode	14	0.98 (0.54- 1.78)	0.95
		Weighted mode	14	1.04 (0.60- 1.79)	0.90
Genus	Howardella	MR Egger	9	0.76 (0.37- 1.57)	0.48
		Weighted median	9	0.96 (0.76- 1.23)	0.76
		Inverse variance weighted	9	1.04 (0.87- 1.24)	0.69
		Simple mode	9	0.93 (0.63- 1.36)	0.72
		Weighted mode	9	0.91 (0.63- 1.32)	0.63
Genus	Hungatella	MR Egger	5	4.56 (0.84- 24.70)	0.18
		Weighted median	5	1.17 (0.80- 1.71)	0.41
		Inverse variance weighted	5	1.10 (0.83- 1.46)	0.51

		Simple mode	5	1.26 (0.70-	2.29)	0.49
		Weighted mode	5	1.30 (0.71-	2.39)	0.44
Genus	Intestinibacter	MR Egger	15	0.88 (0.37-	2.07)	0.77
		Weighted median	15	0.94 (0.66-	1.35)	0.74
		Inverse variance weighted	15	0.94 (0.72-	1.22)	0.64
		Simple mode	15	0.99 (0.53-	1.83)	0.97
		Weighted mode	15	1.01 (0.55-	1.86)	0.97
Genus	Intestinimonas	MR Egger	16	1.25 (0.62-	2.51)	0.54
		Weighted median	16	1.14 (0.81-	1.60)	0.44
		Inverse variance weighted	16	1.00 (0.78-	1.29)	0.99
		Simple mode	16	1.18 (0.70-	2.01)	0.54
		Weighted mode	16	1.15 (0.69-	1.93)	0.60
Genus	Lachnoclostridium	MR Egger	6	1.05 (0.43-	2.60)	0.91
		Weighted median	6	1.09 (0.70-	1.71)	0.70
		Inverse variance weighted	6	1.21 (0.86-	1.70)	0.27
		Simple mode	6	1.05 (0.59-	1.90)	0.87

		Weighted mode	6	1.06 (0.60- 1.88)	0.84
Genus	Lachnospira	MR Egger	6	5.25 (0.02-1103.36)	0.58
		Weighted median	6	0.95 (0.47- 1.93)	0.89
		Inverse variance weighted	6	0.82 (0.35- 1.93)	0.65
		Simple mode	6	1.33 (0.44- 3.96)	0.63
		Weighted mode	6	1.06 (0.38- 2.94)	0.91
Genus	Lachnospiraceae (FCS020 group)	MR Egger	16	1.06 (0.43- 2.60)	0.91
		Weighted median	16	1.00 (0.68- 1.47)	1.00
		Inverse variance weighted	16	0.98 (0.71- 1.35)	0.89
		Simple mode	16	0.89 (0.43- 1.84)	0.75
		Weighted mode	16	0.87 (0.43- 1.75)	0.70
Genus	Lachnospiraceae (NC2004 group)	MR Egger	9	0.73 (0.16- 3.36)	0.69
		Weighted median	9	1.10 (0.77- 1.58)	0.61
		Inverse variance weighted	9	1.08 (0.76- 1.55)	0.66
		Simple mode	9	1.12 (0.67- 1.86)	0.68
		Weighted mode	9	1.12 (0.70- 1.79)	0.65

Genus	Lachnospiraceae (ND3007 group)	MR Egger	3	0.00 (0.00- 0.07)	0.25
		Weighted median	3	1.98 (0.80- 4.91)	0.14
		Inverse variance weighted	3	1.15 (0.34- 3.93)	0.82
		Simple mode	3	2.06 (0.66- 6.44)	0.34
		Weighted mode	3	2.06 (0.69- 6.16)	0.33
Genus	Lachnospiraceae (NK4A136 group)	MR Egger	15	1.10 (0.56- 2.16)	0.79
		Weighted median	15	1.32 (0.88- 1.97)	0.18
		Inverse variance weighted	15	1.04 (0.75- 1.44)	0.83
		Simple mode	15	1.19 (0.64- 2.22)	0.58
		Weighted mode	15	1.28 (0.80- 2.03)	0.32
Genus	Lachnospiraceae (UCG001)	MR Egger	12	1.40 (0.47- 4.15)	0.56
		Weighted median	12	1.13 (0.79- 1.62)	0.50
		Inverse variance weighted	12	1.09 (0.84- 1.41)	0.52
		Simple mode	12	0.95 (0.50- 1.79)	0.87
		Weighted mode	12	0.95 (0.54- 1.68)	0.87
Genus	Lachnospiraceae (UCG004)	MR Egger	12	1.35 (0.28- 6.64)	0.72

		Weighted median	12	1.12 (0.70- 1.80)	0.63
		Inverse variance weighted	12	1.23 (0.85- 1.77)	0.28
		Simple mode	12	0.95 (0.40- 2.28)	0.92
		Weighted mode	12	0.98 (0.42- 2.28)	0.96
Genus	Lachnospiraceae (UCG008)	MR Egger	10	0.75 (0.14- 3.87)	0.74
		Weighted median	10	0.66 (0.47- 0.93)	1.8e-02
		Inverse variance weighted	10	0.74 (0.55- 1.00)	0.05
		Simple mode	10	0.59 (0.31- 1.14)	0.15
		Weighted mode	10	0.59 (0.33- 1.04)	0.10
Genus	Lachnospiraceae (UCG010)	MR Egger	10	0.46 (0.13- 1.67)	0.27
		Weighted median	10	1.22 (0.73- 2.05)	0.44
		Inverse variance weighted	10	1.12 (0.72- 1.76)	0.61
		Simple mode	10	1.24 (0.53- 2.93)	0.63
		Weighted mode	10	1.25 (0.59- 2.65)	0.57
Genus	Lactobacillus	MR Egger	8	1.25 (0.67- 2.33)	0.51
		Weighted median	8	0.89 (0.63- 1.24)	0.49

		Inverse variance weighted	8	0.95 (0.74- 1.20)	0.65
		Simple mode	8	0.78 (0.50- 1.20)	0.29
		Weighted mode	8	0.85 (0.56- 1.27)	0.45
Genus	Lactococcus	MR Egger	9	0.85 (0.33- 2.15)	0.74
		Weighted median	9	1.05 (0.80- 1.37)	0.75
		Inverse variance weighted	9	1.09 (0.88- 1.34)	0.42
		Simple mode	9	1.00 (0.67- 1.50)	0.99
		Weighted mode	9	1.01 (0.65- 1.56)	0.96
Genus	Marvinbryantia	MR Egger	10	2.00 (0.37- 10.88)	0.45
		Weighted median	10	0.91 (0.56- 1.49)	0.72
		Inverse variance weighted	10	0.98 (0.64- 1.51)	0.94
		Simple mode	10	0.89 (0.44- 1.78)	0.74
		Weighted mode	10	0.89 (0.48- 1.62)	0.70
Genus	Methanobrevibacter	MR Egger	6	0.96 (0.37- 2.47)	0.94
		Weighted median	6	1.13 (0.81- 1.58)	0.47
		Inverse variance weighted	6	0.97 (0.76- 1.25)	0.83

		Simple mode	6	1.16 (0.73- 1.84)	0.57
		Weighted mode	6	1.15 (0.73- 1.81)	0.56
Genus	Odoribacter	MR Egger	7	0.34 (0.09- 1.25)	0.17
		Weighted median	7	1.05 (0.60- 1.83)	0.87
		Inverse variance weighted	7	0.90 (0.58- 1.38)	0.63
		Simple mode	7	1.16 (0.48- 2.83)	0.75
		Weighted mode	7	1.10 (0.48- 2.53)	0.82
Genus	Olsenella	MR Egger	10	0.98 (0.45- 2.13)	0.96
		Weighted median	10	0.99 (0.77- 1.27)	0.94
		Inverse variance weighted	10	0.99 (0.79- 1.25)	0.95
		Simple mode	10	1.01 (0.68- 1.48)	0.98
		Weighted mode	10	0.98 (0.73- 1.33)	0.92
Genus	Oscillibacter	MR Egger	13	2.65 (0.68- 10.38)	0.19
		Weighted median	13	1.04 (0.73- 1.47)	0.84
		Inverse variance weighted	13	0.84 (0.58- 1.24)	0.39
		Simple mode	13	1.13 (0.68- 1.85)	0.65

		Weighted mode	13	1.12 (0.70-	1.77)	0.65
Genus	Oscillospira	MR Egger	8	0.51 (0.13-	2.07)	0.39
		Weighted median	8	1.05 (0.68-	1.62)	0.82
		Inverse variance weighted	8	0.91 (0.65-	1.26)	0.56
		Simple mode	8	1.06 (0.56-	2.03)	0.86
		Weighted mode	8	1.06 (0.56-	2.02)	0.85
Genus	Oxalobacter	MR Egger	11	1.10 (0.46-	2.64)	0.83
		Weighted median	11	0.92 (0.72-	1.16)	0.47
		Inverse variance weighted	11	0.93 (0.77-	1.12)	0.46
		Simple mode	11	0.92 (0.65-	1.32)	0.68
		Weighted mode	11	0.92 (0.65-	1.31)	0.65
Genus	Parabacteroides	MR Egger	5	0.35 (0.01-	9.13)	0.57
		Weighted median	5	1.09 (0.59-	2.01)	0.79
		Inverse variance weighted	5	1.17 (0.69-	1.98)	0.55
		Simple mode	5	1.06 (0.46-	2.43)	0.90
		Weighted mode	5	1.02 (0.45-	2.31)	0.97

Genus	Paraprevotella	MR Egger	13	0.94 (0.32-	2.82)	0.92
		Weighted median	13	0.79 (0.59-	1.05)	0.10
		Inverse variance weighted	13	1.10 (0.83-	1.45)	0.52
		Simple mode	13	0.77 (0.50-	1.17)	0.24
		Weighted mode	13	0.77 (0.52-	1.15)	0.22
Genus	Parasutterella	MR Egger	14	0.79 (0.40-	1.58)	0.52
		Weighted median	14	0.83 (0.59-	1.17)	0.28
		Inverse variance weighted	14	0.91 (0.71-	1.17)	0.47
		Simple mode	14	0.78 (0.46-	1.30)	0.35
		Weighted mode	14	0.82 (0.51-	1.32)	0.44
Genus	Peptococcus	MR Egger	12	1.08 (0.41-	2.81)	0.88
		Weighted median	12	1.11 (0.82-	1.48)	0.50
		Inverse variance weighted	12	1.12 (0.89-	1.43)	0.33
		Simple mode	12	0.93 (0.57-	1.52)	0.79
		Weighted mode	12	0.99 (0.61-	1.60)	0.97
Genus	Phascolarctobacterium	MR Egger	8	1.80 (0.35-	9.21)	0.51

		Weighted median	8	0.99 (0.64- 1.54)	0.96
		Inverse variance weighted	8	0.95 (0.67- 1.34)	0.75
		Simple mode	8	0.99 (0.51- 1.91)	0.97
		Weighted mode	8	1.07 (0.58- 1.99)	0.84
Genus	Prevotella 7	MR Egger	11	0.47 (0.10- 2.20)	0.36
		Weighted median	11	0.91 (0.70- 1.19)	0.50
		Inverse variance weighted	11	0.89 (0.68- 1.15)	0.36
		Simple mode	11	0.85 (0.57- 1.27)	0.45
		Weighted mode	11	0.89 (0.61- 1.32)	0.59
Genus	Prevotella 9	MR Egger	15	0.97 (0.42- 2.26)	0.95
		Weighted median	15	0.91 (0.65- 1.27)	0.57
		Inverse variance weighted	15	0.81 (0.61- 1.08)	0.15
		Simple mode	15	0.95 (0.49- 1.85)	0.88
		Weighted mode	15	0.94 (0.49- 1.79)	0.84
Genus	Rikenellaceae (RC9gut group)	MR Egger	11	0.68 (0.24- 1.96)	0.50
		Weighted median	11	0.92 (0.74- 1.14)	0.44

		Inverse variance weighted	11	0.91 (0.77- 1.08)	0.30
		Simple mode	11	0.94 (0.65- 1.37)	0.77
		Weighted mode	11	0.94 (0.65- 1.37)	0.77
Genus	Romboutsia	MR Egger	13	1.24 (0.51- 3.00)	0.64
		Weighted median	13	0.90 (0.60- 1.36)	0.63
		Inverse variance weighted	13	0.99 (0.73- 1.34)	0.93
		Simple mode	13	0.74 (0.34- 1.57)	0.44
		Weighted mode	13	0.79 (0.38- 1.61)	0.52
Genus	Roseburia	MR Egger	14	2.25 (0.80- 6.31)	0.15
		Weighted median	14	1.18 (0.75- 1.85)	0.48
		Inverse variance weighted	14	1.06 (0.76- 1.49)	0.72
		Simple mode	14	1.32 (0.59- 2.96)	0.51
		Weighted mode	14	1.39 (0.63- 3.05)	0.43
Genus	Ruminiclostridium 5	MR Egger	11	0.92 (0.11- 7.83)	0.94
		Weighted median	11	0.73 (0.42- 1.27)	0.26
		Inverse variance weighted	11	0.70 (0.43- 1.14)	0.15

		Simple mode	11	0.61 (0.23- 1.61)	0.35
		Weighted mode	11	0.65 (0.29- 1.46)	0.32
Genus	Ruminiclostridium 6	MR Egger	14	0.94 (0.38- 2.34)	0.89
		Weighted median	14	0.84 (0.55- 1.28)	0.42
		Inverse variance weighted	14	1.03 (0.72- 1.48)	0.87
		Simple mode	14	0.79 (0.41- 1.51)	0.49
		Weighted mode	14	0.81 (0.44- 1.48)	0.50
Genus	Ruminiclostridium 9	MR Egger	7	2.44 (0.09-63.37)	0.61
		Weighted median	7	1.81 (0.91- 3.60)	0.09
		Inverse variance weighted	7	1.51 (0.78- 2.91)	0.22
		Simple mode	7	2.74 (0.82- 9.10)	0.15
		Weighted mode	7	2.60 (0.67-10.05)	0.21
Genus	Ruminococcaceae (NK4A214 group)	MR Egger	13	0.49 (0.15- 1.57)	0.25
		Weighted median	13	1.00 (0.63- 1.59)	0.99
		Inverse variance weighted	13	0.86 (0.60- 1.22)	0.39
		Simple mode	13	1.20 (0.52- 2.74)	0.68

		Weighted mode	13	1.14 (0.43- 3.04)	0.79
Genus	Ruminococcaceae (UCG002)	MR Egger	19	1.21 (0.63- 2.32)	0.57
		Weighted median	19	1.13 (0.80- 1.60)	0.47
		Inverse variance weighted	19	1.06 (0.82- 1.37)	0.66
		Simple mode	19	1.48 (0.79- 2.78)	0.24
		Weighted mode	19	1.23 (0.74- 2.03)	0.44
Genus	Ruminococcaceae (UCG003)	MR Egger	12	1.72 (0.61- 4.87)	0.33
		Weighted median	12	0.82 (0.53- 1.27)	0.37
		Inverse variance weighted	12	0.93 (0.68- 1.29)	0.68
		Simple mode	12	0.61 (0.27- 1.37)	0.26
		Weighted mode	12	0.61 (0.27- 1.37)	0.26
Genus	Ruminococcaceae (UCG004)	MR Egger	11	0.63 (0.13- 2.98)	0.57
		Weighted median	11	1.17 (0.82- 1.68)	0.39
		Inverse variance weighted	11	1.21 (0.92- 1.60)	0.18
		Simple mode	11	1.13 (0.68- 1.90)	0.64
		Weighted mode	11	1.15 (0.70- 1.90)	0.59

Genus	Ruminococcaceae (UCG005)	MR Egger	14	0.80 (0.36- 1.80)	0.60
		Weighted median	14	1.04 (0.70- 1.56)	0.84
		Inverse variance weighted	14	1.18 (0.87- 1.58)	0.29
		Simple mode	14	1.22 (0.61- 2.47)	0.58
		Weighted mode	14	1.08 (0.64- 1.84)	0.78
Genus	Ruminococcaceae (UCG009)	MR Egger	12	0.79 (0.31- 2.00)	0.63
		Weighted median	12	0.99 (0.71- 1.37)	0.94
		Inverse variance weighted	12	0.94 (0.75- 1.18)	0.60
		Simple mode	12	1.03 (0.57- 1.85)	0.92
		Weighted mode	12	1.03 (0.60- 1.76)	0.91
Genus	Ruminococcaceae (UCG010)	MR Egger	6	1.01 (0.33- 3.05)	0.99
		Weighted median	6	0.94 (0.56- 1.58)	0.83
		Inverse variance weighted	6	0.90 (0.60- 1.34)	0.60
		Simple mode	6	0.95 (0.48- 1.87)	0.88
		Weighted mode	6	0.95 (0.50- 1.81)	0.88
Genus	Ruminococcaceae (UCG011)	MR Egger	8	2.20 (0.83- 5.81)	0.16

		Weighted median	8	1.06 (0.82- 1.38)	0.66
		Inverse variance weighted	8	0.98 (0.81- 1.19)	0.83
		Simple mode	8	1.13 (0.75- 1.70)	0.57
		Weighted mode	8	1.15 (0.79- 1.68)	0.49
Genus	Ruminococcaceae (UCG013)	MR Egger	11	0.56 (0.21- 1.52)	0.28
		Weighted median	11	1.07 (0.66- 1.74)	0.77
		Inverse variance weighted	11	1.12 (0.79- 1.58)	0.54
		Simple mode	11	1.07 (0.48- 2.40)	0.87
		Weighted mode	11	1.07 (0.53- 2.19)	0.85
Genus	Ruminococcaceae (UCG014)	MR Egger	11	1.13 (0.49- 2.59)	0.79
		Weighted median	11	1.18 (0.77- 1.81)	0.45
		Inverse variance weighted	11	1.24 (0.88- 1.73)	0.22
		Simple mode	11	1.15 (0.66- 2.00)	0.63
		Weighted mode	11	1.18 (0.76- 1.84)	0.48
Genus	Ruminococcus 1	MR Egger	10	1.46 (0.58- 3.67)	0.44
		Weighted median	10	0.89 (0.56- 1.41)	0.61

		Inverse variance weighted	10	0.85 (0.60- 1.20)	0.36
		Simple mode	10	0.78 (0.37- 1.66)	0.54
		Weighted mode	10	0.85 (0.43- 1.67)	0.65
Genus	Ruminococcus 2	MR Egger	15	0.52 (0.27- 1.04)	0.09
		Weighted median	15	0.72 (0.49- 1.06)	0.10
		Inverse variance weighted	15	0.80 (0.60- 1.07)	0.13
		Simple mode	15	0.82 (0.43- 1.54)	0.54
		Weighted mode	15	0.69 (0.41- 1.15)	0.17
Genus	Sellimonas	MR Egger	13	1.24 (0.51- 3.00)	0.64
		Weighted median	13	0.90 (0.60- 1.36)	0.63
		Inverse variance weighted	13	0.99 (0.73- 1.34)	0.93
		Simple mode	13	0.74 (0.36- 1.50)	0.41
		Weighted mode	13	0.79 (0.38- 1.61)	0.52
Genus	Senegalimassilia	MR Egger	5	0.57 (0.13- 2.51)	0.51
		Weighted median	5	0.85 (0.52- 1.38)	0.51
		Inverse variance weighted	5	0.87 (0.59- 1.27)	0.46

		Simple mode	5	0.79 (0.41- 1.51)	0.52
		Weighted mode	5	0.74 (0.37- 1.50)	0.46
Genus	Slackia	MR Egger	11	0.92 (0.11- 7.83)	0.94
		Weighted median	11	0.73 (0.43- 1.25)	0.25
		Inverse variance weighted	11	0.70 (0.43- 1.14)	0.15
		Simple mode	11	0.61 (0.24- 1.54)	0.32
		Weighted mode	11	0.65 (0.27- 1.54)	0.35
Genus	Streptococcus	MR Egger	12	0.49 (0.08- 2.90)	0.45
		Weighted median	12	0.81 (0.50- 1.31)	0.39
		Inverse variance weighted	12	0.84 (0.53- 1.35)	0.47
		Simple mode	12	0.83 (0.40- 1.73)	0.62
		Weighted mode	12	0.81 (0.44- 1.50)	0.52
Genus	Subdoligranulum	MR Egger	11	0.67 (0.28- 1.61)	0.39
		Weighted median	11	0.58 (0.36- 0.95)	3.2e-02
		Inverse variance weighted	11	0.61 (0.44- 0.86)	5.1e-03
		Simple mode	11	0.56 (0.28- 1.13)	0.14

		Weighted mode	11	0.55 (0.27- 1.13)	0.13
Genus	Sutterella	MR Egger	12	1.08 (0.16- 7.12)	0.94
		Weighted median	12	0.89 (0.56- 1.42)	0.63
		Inverse variance weighted	12	0.91 (0.60- 1.38)	0.66
		Simple mode	12	0.83 (0.39- 1.77)	0.64
		Weighted mode	12	0.93 (0.48- 1.79)	0.82
Genus	Terrisporobacter	MR Egger	5	0.85 (0.08- 8.62)	0.90
		Weighted median	5	0.60 (0.35- 1.04)	0.07
		Inverse variance weighted	5	0.81 (0.42- 1.58)	0.54
		Simple mode	5	0.51 (0.21- 1.21)	0.20
		Weighted mode	5	0.51 (0.24- 1.11)	0.17
Genus	Turicibacter	MR Egger	9	0.96 (0.29- 3.15)	0.94
		Weighted median	9	0.89 (0.61- 1.30)	0.55
		Inverse variance weighted	9	0.90 (0.67- 1.21)	0.50
		Simple mode	9	0.95 (0.52- 1.72)	0.86
		Weighted mode	9	0.89 (0.51- 1.54)	0.68

Genus	Tyzzerella 3	MR Egger	13	1.15 (0.37- 3.51)	0.81
		Weighted median	13	1.21 (0.92- 1.59)	0.17
		Inverse variance weighted	13	1.15 (0.95- 1.40)	0.16
		Simple mode	13	1.24 (0.78- 1.96)	0.39
		Weighted mode	13	1.23 (0.81- 1.87)	0.35
Genus	Unknown genus(826)	MR Egger	15	1.39 (0.72- 2.69)	0.35
		Weighted median	15	1.14 (0.80- 1.62)	0.47
		Inverse variance weighted	15	1.21 (0.95- 1.53)	0.13
		Simple mode	15	1.22 (0.72- 2.08)	0.47
		Weighted mode	15	1.17 (0.73- 1.87)	0.53
Genus	Unknown genus (959)	MR Egger	9	1.42 (0.69- 2.95)	0.37
		Weighted median	9	1.27 (0.92- 1.76)	0.15
		Inverse variance weighted	9	1.14 (0.89- 1.46)	0.29
		Simple mode	9	1.31 (0.83- 2.08)	0.28
		Weighted mode	9	1.32 (0.88- 2.00)	0.22
Genus	Unknown genus (1868)	MR Egger	12	1.77 (0.64- 4.89)	0.30

		Weighted median	12	1.03 (0.69- 1.54)	0.87
		Inverse variance weighted	12	1.06 (0.75- 1.48)	0.75
		Simple mode	12	1.03 (0.52- 2.05)	0.92
		Weighted mode	12	1.03 (0.54- 1.95)	0.94
Genus	Unknown genus (2001)	MR Egger	8	1.28 (0.26- 6.27)	0.77
		Weighted median	8	1.17 (0.79- 1.74)	0.43
		Inverse variance weighted	8	1.17 (0.81- 1.69)	0.40
		Simple mode	8	1.12 (0.54- 2.32)	0.76
		Weighted mode	8	1.15 (0.61- 2.15)	0.68
Genus	Unknown genus (2041)	MR Egger	12	0.66 (0.30- 1.44)	0.32
		Weighted median	12	0.88 (0.68- 1.13)	0.31
		Inverse variance weighted	12	0.94 (0.78- 1.14)	0.55
		Simple mode	12	0.85 (0.58- 1.25)	0.44
		Weighted mode	12	0.84 (0.57- 1.22)	0.38
Genus	Unknown genus(2071)	MR Egger	10	1.79 (0.67- 4.73)	0.28
		Weighted median	10	1.01 (0.68- 1.49)	0.96

		Inverse variance weighted	10	0.94 (0.70- 1.27)	0.69
		Simple mode	10	0.98 (0.51- 1.87)	0.95
		Weighted mode	10	0.98 (0.52- 1.83)	0.95
Genus	Unknown genus (2755)	MR Egger	9	1.35 (0.59- 3.10)	0.50
		Weighted median	9	1.26 (0.85- 1.86)	0.25
		Inverse variance weighted	9	1.15 (0.87- 1.53)	0.33
		Simple mode	9	1.37 (0.79- 2.37)	0.29
		Weighted mode	9	1.29 (0.83- 2.00)	0.29
Genus	Unknown genus(1000000073)	MR Egger	12	0.76 (0.31- 1.87)	0.56
		Weighted median	12	1.07 (0.77- 1.50)	0.69
		Inverse variance weighted	12	1.14 (0.86- 1.50)	0.36
		Simple mode	12	1.00 (0.58- 1.72)	0.99
		Weighted mode	12	1.05 (0.64- 1.73)	0.85
Genus	Unknown genus (1000001215)	MR Egger	15	1.40 (0.31- 6.38)	0.67
		Weighted median	15	0.95 (0.65- 1.40)	0.81
		Inverse variance weighted	15	1.07 (0.80- 1.43)	0.67

		Simple mode	15	0.92 (0.45- 1.85)	0.81
		Weighted mode	15	0.96 (0.51- 1.83)	0.91
Genus	Unknown genus(1000005472)	MR Egger	13	0.76 (0.30- 1.90)	0.57
		Weighted median	13	0.84 (0.61- 1.17)	0.30
		Inverse variance weighted	13	0.88 (0.70- 1.12)	0.31
		Simple mode	13	0.68 (0.38- 1.23)	0.23
		Weighted mode	13	0.82 (0.49- 1.37)	0.46
Genus	Unknown genus (1000005479)	MR Egger	13	1.07 (0.48- 2.38)	0.87
		Weighted median	13	0.90 (0.62- 1.31)	0.58
		Inverse variance weighted	13	0.92 (0.70- 1.22)	0.56
		Simple mode	13	0.90 (0.49- 1.65)	0.74
		Weighted mode	13	0.95 (0.53- 1.69)	0.86
Genus	Unknown genus (1000006162)	MR Egger	11	1.40 (0.33- 5.89)	0.66
		Weighted median	11	1.17 (0.87- 1.56)	0.30
		Inverse variance weighted	11	1.01 (0.77- 1.33)	0.92
		Simple mode	11	1.29 (0.83- 2.00)	0.29

		Weighted mode	11	1.22 (0.79- 1.89)	0.38
Genus	Veillonella	MR Egger	6	1.71 (0.08-37.76)	0.75
		Weighted median	6	0.87 (0.53- 1.44)	0.60
		Inverse variance weighted	6	0.70 (0.48- 1.02)	0.06
		Simple mode	6	0.93 (0.46- 1.88)	0.85
		Weighted mode	6	0.93 (0.47- 1.81)	0.83
Genus	Victivallis	MR Egger	10	0.40 (0.09- 1.82)	0.27
		Weighted median	10	0.98 (0.76- 1.27)	0.88
		Inverse variance weighted	10	0.96 (0.78- 1.17)	0.66
		Simple mode	10	1.21 (0.76- 1.94)	0.45
		Weighted mode	10	1.20 (0.75- 1.93)	0.46

Abbreviations: MR, Mendelian randomization study; SS, Sjögren's syndrome; SNP, single nucleotide polymorphism; OR, odds ratio.

Table S3 The heterogeneity of gut microbiota instrumental variables.

Group	Gut microbiota	Cochran's Q	df	P-value
Phylum	Lentisphaerae	8.34	8	0.401
Class	Deltaproteobacteria	16.33	12	0.177
Family	Porphyromonadaceae	8.88	11	0.633
Genus	Eubacterium coprostanoligenes group	3.58	7	0.826
Genus	Blautia	8.85	9	0.451
Genus	Butyricicoccus	5.54	10	0.852
Genus	Escherichia.Shigella	6.90	8	0.546
Genus	Subdoligranulum	9.30	10	0.504

Table S4 Directional horizontal pleiotropy assessed by intercept term in MR Egger regression of the association between gut microbiota and SS.

Group	Gut microbiota	Egger_intercept	SE	P-value
Phylum	Lentisphaerae	0.07	0.06	0.272
Class	Deltaproteobacteria	0.08	0.05	0.132
Family	Porphyromonadaceae	-0.05	0.03	0.179
Genus	Eubacterium coprostanoligenes group	0.02	0.03	0.478
Genus	Blautia	-0.02	0.04	0.724
Genus	Butyricicoccus	-0.007	0.03	0.843
Genus	Escherichia.Shigella	0.08	0.06	0.211
Genus	Subdoligranulum	-0.02	0.06	0.708

Table S5 MR-PRESSO analysis for the association between gut microbiota and SS.

Group	Gut microbiota	Causal Estimate	SD	T	Global test P-value
Phylum	Lentisphaerae	-0.20	0.11	-1.94	0.402
Class	Deltaproteobacteria	-0.36	0.17	-2.17	0.574
Family	Porphyromonadaceae	-0.36	0.22	-1.62	0.383
Genus	Eubacterium coprostanoligenes group	-0.49	0.21	-2.30	0.199
Genus	Blautia	-0.47	0.17	-2.78	0.663
Genus	Butyricicoccus	-0.38	0.20	-1.86	0.416
Genus	Escherichia.Shigella	-0.28	0.12	-2.36	0.785
Genus	Subdoligranulum	-0.38	0.12	-3.12	0.855
