# Supplementary figures and images for: Causal Relationship Between Sjögren’s Syndrome and Gut Microbiota: A Two-Sample Mendelian Randomization Study
Source: Biomedicines. 2024 Oct 18;12(10):2378. doi: 10.3390/biomedicines12102378 (PMC11505323; doi:10.3390/biomedicines12102378)

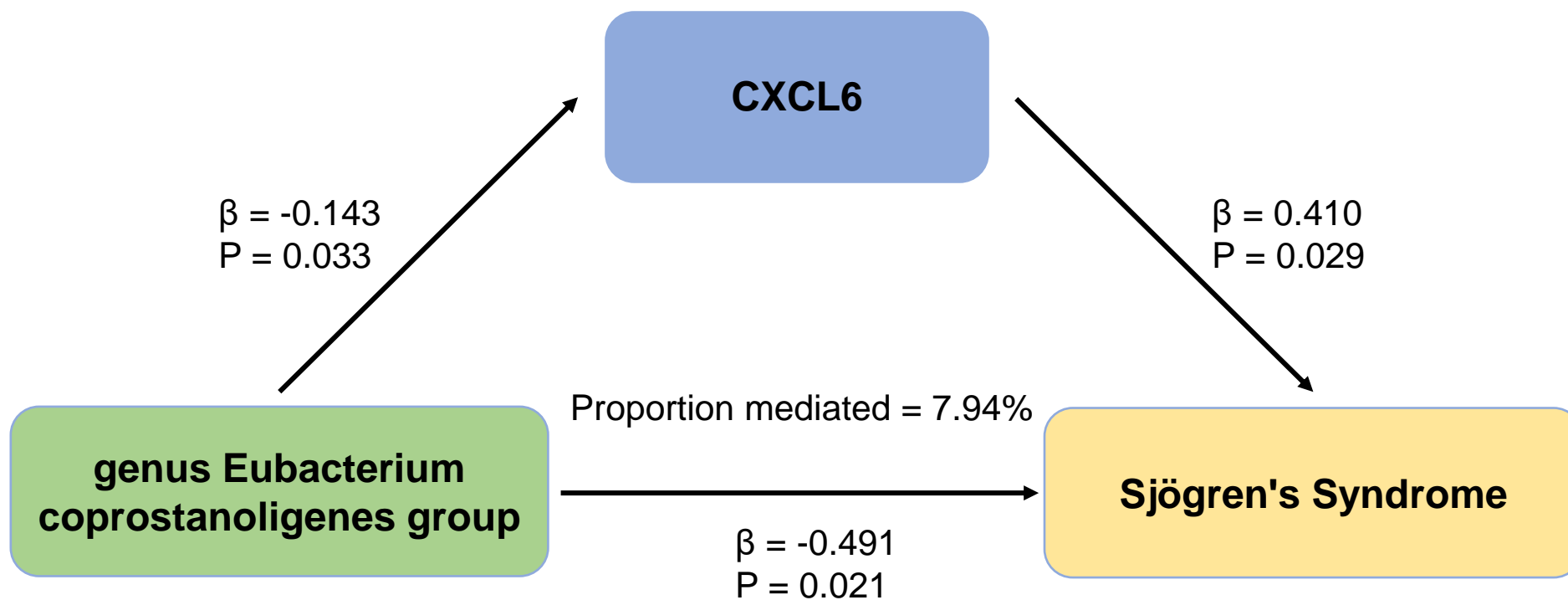

Supplement: Supplementary file 1 [file biomedicines-12-02378-s001.zip › Supplementary Figure S1.pdf]
